# Implications of CI therapy for visual deficit training

**DOI:** 10.3389/fnint.2014.00078

**Published:** 2014-10-09

**Authors:** Edward Taub, Victor W. Mark, Gitendra Uswatte

**Affiliations:** University of Alabama at BirminghamBirmingham, AL, USA

**Keywords:** CI therapy, neuroplasticity, neurorehabilitation, motor deficits, visual deficits, aphasia, learned nonuse, sensory nonuse

## Abstract

We address here the question of whether the techniques of Constraint Induced (CI) therapy, a family of treatments that has been employed in the rehabilitation of movement and language after brain damage might apply to the rehabilitation of such visual deficits as unilateral spatial neglect and visual field deficits. CI therapy has been used successfully for the upper and lower extremities after chronic stroke, cerebral palsy (CP), multiple sclerosis (MS), other central nervous system (CNS) degenerative conditions, resection of motor areas of the brain, focal hand dystonia, and aphasia. Treatments making use of similar methods have proven efficacious for amblyopia. The CI therapy approach consists of four major components: intensive training, training by shaping, a “transfer package” to facilitate the transfer of gains from the treatment setting to everyday activities, and strong discouragement of compensatory strategies. CI therapy is said to be effective because it overcomes learned nonuse, a learned inhibition of movement that follows injury to the CNS. In addition, CI therapy produces substantial increases in the gray matter of motor areas on both sides of the brain. We propose here that these mechanisms are examples of more general processes: learned nonuse being considered parallel to sensory nonuse following damage to sensory areas of the brain, with both having in common diminished neural connections (DNCs) in the nervous system as an underlying mechanism. CI therapy would achieve its therapeutic effect by strengthening the DNCs. Use-dependent cortical reorganization is considered to be an example of the more general neuroplastic mechanism of brain structure repurposing. If the mechanisms involved in these broader categories are involved in each of the deficits being considered, then it may be the principles underlying efficacious treatment in each case may be similar. The lessons learned during CI therapy research might then prove useful for the treatment of visual deficits.

## INTRODUCTION

In a previous article it was suggested that the principles that underlie the rehabilitation of deficits in movement and speech by Constraint Induced (CI) therapy training are similar to the improvement by training of visual deficits in amblyopia (Taub, 2010). Both types of remediation probably have one mechanism in common, overcoming learned nonuse, and perhaps another, neuroplastic change. In the present article, the possibility will be raised that similar principles may underlie efficacious rehabilitation training to improve other visual deficits and perhaps deficits in other sensory systems, such as somatic sensation.

In the first half of the article the origin, methods, and representative results of CI movement therapy will be described, as well as the extension of this rehabilitation method from motor deficit after stroke to motor deficit after traumatic brain injury (TBI), cerebral palsy, and multiple sclerosis (MS). The extension of CI therapy from stroke to these other conditions was based, actually predicted, by the learned nonuse formulation. The application of the CI therapy approach, again based on the learned nonuse formulation, to a non-motor neurological disorder, post-stroke aphasia, will be presented. This will be followed by an analysis of amblyopia training, outlining the detailed similarity of methodology and type of results obtained with this efficacious treatment for a sensory deficit to those of CI therapy. Next the research on training techniques used for the rehabilitation of two common visual deficits resulting from brain damage, unilateral spatial neglect, and visual field defects, will be summarized briefly. Given the prior effectiveness of CI therapy with two non-motor deficits, aphasia and particularly the visual deficit associated with amblyopia, it will be suggested that the application of a CI therapy approach to unilateral neglect and visual field defects might enhance the impressive effects that have been achieved in their treatment to date.

In the past, evidence has been presented to indicate that at least two mechanisms underlie the operation of CI therapy. One mechanism, use-dependent neuroplastic alteration in cortical territorial function, would presumably remain the same for both CI motor therapy/CI aphasia therapy and the rehabilitation of visual deficits. The concept of learned nonuse, however, would have to be broadened to include “sensory nonuse,” both learned motor nonuse and sensory nonuse having in common the development of diminished neural connections (DNCs) after damage to the brain, or nonuse of a function, or both; their rehabilitation then could be referred to as DNC strengthening. Importantly, the concept of DNC strengthening provides a bridge connecting overcoming learned nonuse/sensory nonuse on the one hand and use-dependent cortical reorganization on the other.

The plan of the article then is to first review CI therapy and then to suggest the potential value of applying the principles and general procedures of the CI therapy protocol to visual deficits. Considerable space is spent on describing CI therapy since that is what is known. It is hoped that the detail of this account can provide a platform upon which appropriate experiments can be designed for developing new treatments for the rehabilitation of visual deficits.

## CI THERAPY

### ORIGIN – PRIMATE STUDIES ON SOMATOSENSORY DEAFFERENTATION

Constraint Induced therapy is derived from basic behavioral neuroscience research. It was an early observation in neuroscience research that when a single forelimb in monkeys is surgically deprived of somatic sensation by serial dorsal rhizotomy, the animal does not make use of the deafferented extremity in the free situation. This phenomenon was observed repeatedly in the history of neuroscience (Mott and Sherrington, 1895; Lassek and Moyer, 1953; Twitchell, 1954) and the nonuse was long thought to be irremediable. However, we found that monkeys can be induced to use their deafferented extremity by one of two behavioral techniques (summarized in Taub, 1977, 1980). One technique is training; the type of training that was found to be particularly effective is termed shaping. Shaping is an operant conditioning training method in which a desired behavioral or other functional objective is approached in small steps, by “successive approximations,” so that the improvement required for successful performance at any one point in the training is small (Skinner, 1938, 1968; Taub et al., 1994). The actions shaped in the primate deafferentation experiment included (a) pointing at visual targets (Taub et al., 1975a) and (b) prehension in juveniles deafferented on day-of-birth (Taub et al., 1973) and prenatally (Taub et al., 1975b) who had never exhibited any prehension previously. The second behavioral technique was restricting movement of the intact limb. The monkey was thereby forced to either use the deafferented extremity or be rendered virtually helpless, unable to ambulate, climb, or grasp food to feed itself. The monkey may not have used the affected extremity for several years, but the application of either of these two techniques resulted in a striking conversion of a useless forelimb into a limb that was used for a wide variety of purposes. The movements were not normal; they were clumsy since somatic sensation had been abolished, but they were extensive and effective. When the experimental manipulations were more than just transient, the reversal was permanent, persisting for the remainder of the animal’s life.

The nonuse of a single deafferented limb had been used by Sherrington, based on his experiment in 1895 with Mott (Mott and Sherrington, 1895), as one of the primary foundations of his influential reflexological position in which reflex mechanisms were viewed as the basic building blocks underlying purposive movement (Sherrington, 1931). Since the monkeys in the above-noted experiments could be induced to make extensive and effective purposive movements with a limb from which all same segment reflex arcs had been abolished, the reflexological explanation of movement could not be correct. To explain the nonuse of an extremity that followed its deafferentation and the subsequent overcoming of that nonuse by one of the two techniques that we employed, the learned nonuse formulation was developed (Taub, 1977, 1980; Taub et al., 2006b). In brief, the monkeys were said to not use the deafferented limb because they learned not to use it in the early postoperative period. The behavioral “contingencies of reinforcement” that established the nonuse condition persisted throughout the lifespan, and thus so did the nonuse of the limb, but this could be overcome by a technique which consistently increased the motivation to use the limb, such as either of the two techniques that we employed. (For a fuller account of this formulation, see “Learned Nonuse” section below). A fundamental aspect of the formulation was that learned nonuse should occur after any substantial damage to the central nervous system (CNS). The formulation was developed to explain the results after single forelimb deafferentation in monkeys and its first application in humans was to stroke. However, from the outset the formulation was meant to apply to deficits that followed any type of damage to the CNS which, depending on the location of the CNS damage, could be motor, visual, or possibly some other sensory system (and to some peripheral somatic injuries as well, e.g., fractured hip, arthritis).

### APPLICATION OF THE REHABILITATION PROTOCOL DEVELOPED WITH DEAFFERENTED MONKEYS TO HUMANS AFTER STROKE

The conversion of a useless extremity to a limb that was used extensively on a permanent basis clearly constituted a strong form of rehabilitation, though the term rehabilitation was not used in relation to animals at the time. When considering the primate results in this light, the application of the techniques employed with primates to the rehabilitation of function after CNS damage in humans seemed plausible. As noted, the first application was to stroke. The original two components used with monkeys were kept basically intact, but the human environment and motivation structure differs substantially from that of primates in an animal colony. Therefore, in making the translation from monkeys to humans two new elements were added. The four components of the treatment that have been used in all of its applications with humans are as follows (Taub, 2004; Morris et al., 2006; Taub and Uswatte, 2006; Taub et al., 2006a): (1) intensive training of the more-affected arm for multiple days; (2) training with the behavioral technique termed shaping; (3) the transfer package (TP), a set of behavioral techniques designed to facilitate transfer of therapeutic gains from the treatment setting to daily life; and (4) discouraging compensatory activities that are employed to avoid using the impaired function. For unilateral upper extremity motor deficit after stroke or other hemiparetic illnesses (as well as somatosensory deafferentation of a limb in monkeys) the intact limb is prevented from doing the work of both arms by restraining it. For aphasia after stroke, communication by gesture or any other non-verbal means is strongly discouraged; there is no physical restraint. There is also no physical restraint used for lower extremity CI therapy.

The training method of shaping involves approaching a behavioral objective in small steps by “successive approximations” (i.e., a task is gradually made more difficult with respect to a participant’s motor capabilities). Its principles were explicitly formulated by Skinner (1938, 1968) and they have been applied to the rehabilitation of movement in this laboratory (Taub et al., 1993, 1994). For rehabilitation, shaping involves (a) providing immediate and very frequent feedback concerning improvements in the speed and quality of movement or speech, (b) selecting tasks that are tailored to address the deficits of individual participants, (c) modeling, prompting, and cuing of task performance, (d) systematically increasing the task difficulty in small steps when improvement is present for a period of time, and (e) motivating the participant to improve performance by what might informally be called “cheerleading.” In this laboratory shaping has two distinct levels: (1) Improving the speed and quality of movement from trial to trial within a task with frequent feedback and encouragement being given. (2) Introducing a new task that is similar to but more difficult than the one being used when motor performance improves to the point where the therapist feels that the new task can be accomplished by the participant. The procedure employed here involves use of both levels of shaping but focuses more attention on improving within-level task performance.

The TP consists of a set of techniques commonly used in the behavior analysis field (Taub, 2012, 2013) to treat a variety of conditions for such problems as medication adherence, adherence to an at-home exercise regimen for low back pain, drug addiction treatment, addiction relapse prevention, and alteration of autism spectrum behaviors; but they have not been used systematically in physical rehabilitation. The TP techniques used here are: behavioral contracts, daily home diary, tracking amount and quality of use of the more-affected arm in 30 important activities of daily living (ADL) in the life situation by daily administration of the Motor Activity Log (MAL), problem solving to overcome perceived barriers to more-affected arm or language use in ADL performance, written assignment during treatment of practice to be carried out at home of use of the more-affected arm in specified ADL (home skill assignment), post-treatment home skill practice assignments, weekly telephone calls for the first month after laboratory treatment in which the MAL is given and problem solving carried out. For further description of the TP techniques and a demonstration of its efficacy see Taub et al. (2013b) and its online supplement.

Taub and coworkers (Taub et al., 1993, 2006a) applied this protocol to the rehabilitation of persons with a chronic upper extremity hemiparesis in two randomized controlled trials (RCTs) that employed attention-placebo control groups and emphasized transfer of therapeutic gains in the laboratory to the life situation. Patients with chronic, rather than acute, stroke were targeted as subjects for this study because in the primate deafferentation research substantial motor rehabilitation was possible well into the chronic phase. In addition, according to the research literature at the time (Twitchell, 1951; Bard and Hirschberg, 1965; Parker et al., 1986), and almost universal clinical experience, spontaneous motor recovery was thought to plateau within 1 year after stroke. There was no evidence that any treatment could produce further recovery of function more than 1 year after stroke. Therefore, any marked improvement in the motor function of individuals with chronic stroke would be of particular therapeutic significance. After a long-standing plateau, the probability would be very low that an abrupt, large improvement in motor ability could be due to spontaneous recovery.

### LARGE DIFFERENTIAL EFFECT OF THE TREATMENT ON (1) IMPAIRMENT AND MOTOR CAPACITY TESTED IN THE LABORATORY AND (2) ACTUAL ADL PERFORMANCE IN THE LIFE SITUATION

**FIGURE 1:**
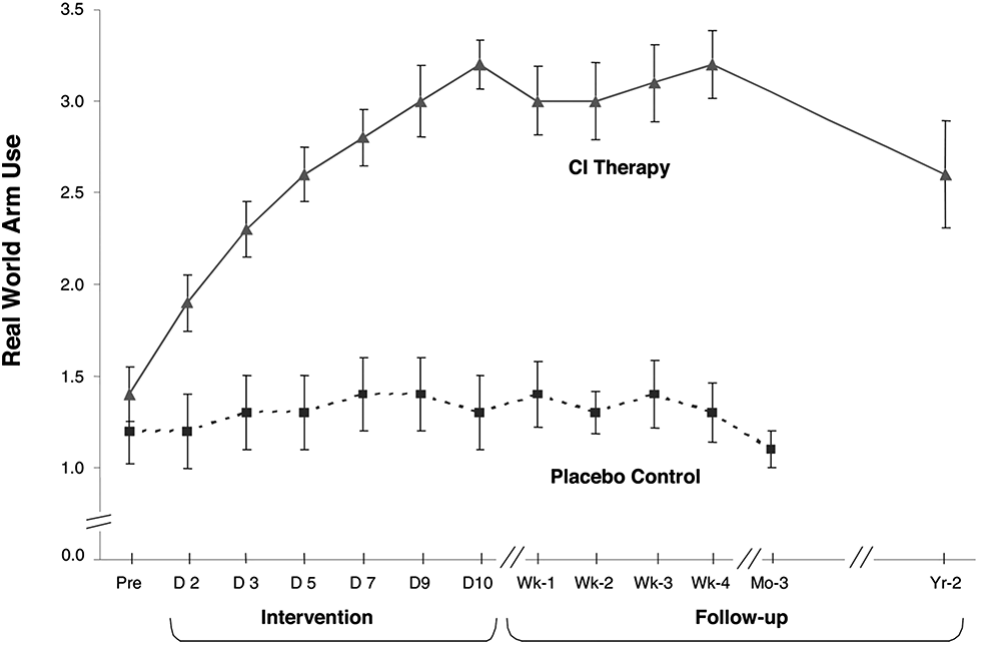
**Mean MAL arm use scores from CI therapy (*n* = 21) and placebo control (*n* = 20) patients with chronic stroke.** CI therapy subjects showed a very large improvement in arm use outside the laboratory from pretreatment to post-treatment (1.8 ± 0.6; *P <* 0.0001; *d’* = 3.0). Before treatment the data indicate that these patients were using the more affected arm 14% as much as before stroke, while after 2 weeks of treatment it was 52%, an almost four times increase. Controls showed little change. CI therapy subjects retained all of their immediate treatment gains 4 weeks after therapy and showed only a 23% decrease after 2 years from post-treatment levels of real-world arm use. At 1-year post-treatment the loss in retention was 14%. Reprinted from Taub et al. (2006a).

Over 1000 adult and pediatric patients with chronic stroke with mild/moderate motor deficits (grade 2; an estimated 25% of the chronic stroke population) have been given upper extremity CI therapy in this laboratory and clinic. On the level of impairment, active range of motion (AROM) was assessed for 26 movements in adults distributed among each of the arm joints. Of the joints whose movements were outside of normal limits prior to treatment, 44% improved more than 5^∘^. The mean improvement for these joints was 22% relative to their pretreatment joint angle value. Motor capacity was measured by the Wolf Motor Function Test (WMFT; Wolf et al., 1989, 2005; Taub et al., 1993; Morris et al., 2001), a laboratory motor function test in which the tester requests that the subjects make the best movements of which they are capable in 15 timed tasks. The effect size (ES) of the mean pre- to post-treatment change was *d′* = 0.9 for this measure. Actual performance of ADL in the life situation was measured by the MAL (Taub et al., 1993, 2006a) a structured, scripted interview with established reliability and validity (van der Lee et al., 2004; Uswatte et al., 2005b, 2006b). The mean ES (*d′*) for the MAL was 3.3. The treatment change for ADL in the life situation from one RCT (Taub et al., 2006a) is shown in **Figure 1**.

The much larger ES for the MAL than for the WMFT indicates that CI therapy has its greatest effect on increasing the actual amount of use of a more-affected upper extremity in the real-world setting, though the improvement in maximal motor capacity as indexed by the WMFT is still substantial. In the meta-analysis literature, an ES (*d′*) of 0.2 is considered small, a 0.4–0.6 *d′* is moderate, while *d′*s of 0.8 and above are large (Cohen, 1988). Thus, the ES of CI therapy for improvement in motor function on a laboratory test where a best effort is requested is large, but for real-world outcome in patients with chronic stroke the ES is extremely large. This differential effect would appear to be due to the ability of CI therapy to overcome the “learned nonuse” that frequently depresses the spontaneous use of a more-affected arm after CNS damage. It should be emphasized, though, that even though CI therapy has its largest effect on ADL function in the real world, it also has a large effect on both the impairment level and on performance on a laboratory motor function test. This will be an important consideration for the applicability of the CI therapy protocol to visual deficits.

### NAMING THE TREATMENT: CONSTRAINT-INDUCED MOVEMENT THERAPY (CI THERAPY)

The movement-restriction and training situations of CI therapy share a common feature. They both are powerful means of inducing use of the more-affected arm. One procedure physically *restrains* the less-affected arm so that the individual must use the more-affected extremity to avoid being rendered more dependent or, in the case of the unilaterally deafferented monkeys, virtually helpless. The other method, training, induces use of the more-affected arm by structuring a situation so that the limb must be used in order to achieve success or avoid failure. Thus, both procedures constitute *constraints* that promote use of the more-affected arm by a major alteration of environmental conditions. Though the name of the treatment is accurate, the use of the term “constraint” in the title of the therapy has turned out to be confusing. The most salient aspect of CI therapy for the upper extremity to a casual observer is that the less-affected arm is *restrained*. Moreover, the rehabilitation field was not used to thinking of training as imposing a constraint on behavior. Instead, the large majority of professionals interpreted the focal word in the name of the therapy as being an alternate way of saying “restraint.” Thus, the general impression arose that restraint of the less-affected arm was the central and most important feature of the therapy. That is very far from being true; physical restraint of the less-affected arm can be dispensed with entirely in achieving a maximal result if the training conditions are arranged appropriately.

Variants of upper extremity CI therapy that do not involve physical restraint of the less affected arm have been found to be as efficacious as the initial protocol (Taub et al., 1996, 1998; Taub and Uswatte, 2000; Uswatte et al., 2006a). These include (1) placement of a non-restrictive half-glove (with fingers cut off) on the less-affected arm as a reminder not to use it and shaping of the paretic arm, and (2) shaping of the paretic arm only (Uswatte et al., 2006a). The half-glove intervention was designed so that CI therapy could be employed with patients who have balance problems and might be at risk for falls when wearing a sling; this intervention expanded the population of stroke patients amenable to CI therapy threefold. Currently, a padded or protective safety mitt is used instead of an arm sling. This restraint leaves the less-affected arm free so that it can be used for defense in case of a fall, but prevents use of the hand and fingers in ADL. Thus, there is nothing talismanic about use of a restraint device. Any rehabilitation technique that requires that the more-affected arm be used extensively should be efficacious.

### IMPORTANCE OF TRANSFERRING TREATMENT GAINS FROM CLINIC TO LIFE SITUATION: ROLE OF THE TRANSFER PACKAGE

In most rehabilitation regimens, participants carry out exercises guided by a therapist during treatment sessions, and then the treatment usually stops. The TP of techniques makes patients more active participants in their own improvement, not only during the treatment sessions but also at home. The TP provides a systematic means of specifying explicitly what the participant is expected to do when outside the treatment setting, monitors what in fact is done, and provides a structure within which to solve apparent barriers to carrying out treatment goals. Thus, the TP immerses participants in a therapeutic environment for a meaningful portion of their day. Therapy is not confined to the limited period that the current health care system with its reimbursement limits permit.

A 2 × 2 factorial experimental components analysis of CI therapy was carried out to assess the relative contribution made by the TP and shaping to the magnitude of the treatment effect (Taub et al., 2013b). Two levels, presence versus absence, of each of these treatment factors, TP and shaping, were tested. Thus, there were four groups: (1) the full CI therapy package, which includes the TP and shaping, (2) the CI therapy package minus the shaping component only, (3) the CI therapy package minus the TP component only, and (4) the CI therapy package minus the shaping and TP components. In other words, all four groups received the same amount and intensity of training and wore a padded mitt preventing use of the more-affected hand during training in the laboratory, but they varied in whether they received training on upper-extremity tasks in the laboratory with shaping or with practice on tasks at the same level of difficulty throughout treatment; they also varied in whether they received the TP or not. Spontaneous use of the more-affected arm in daily life and maximum motor capacity of that arm in the laboratory were assessed with the MAL and the WMFT, respectively.

**FIGURE 2:**
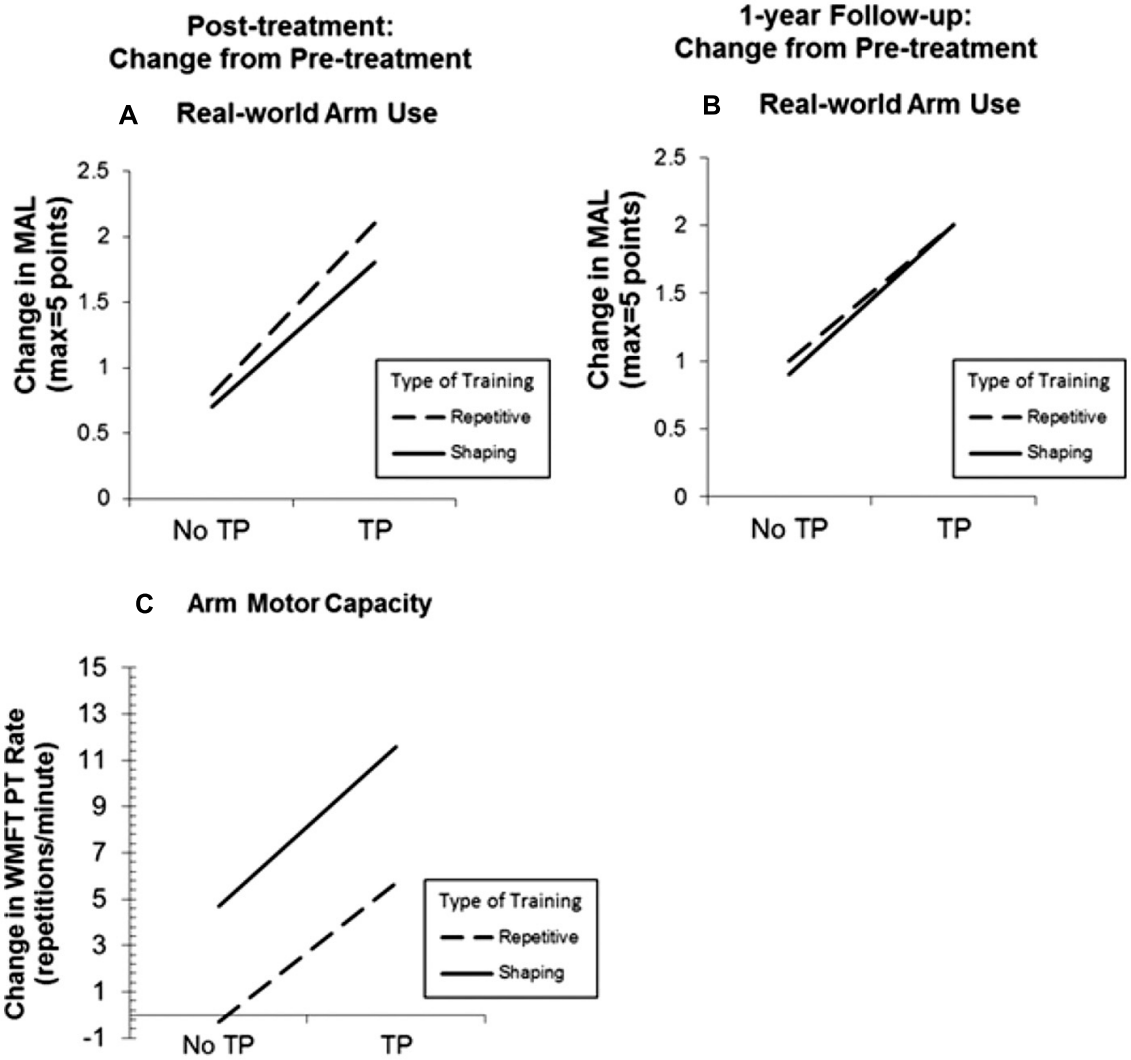
**Treatment outcome increase resulting from administration of the transfer package (TP) and shaping for real-world spontaneous use of more-affected arm (Motor Activity Log, MAL) and the maximum motor capacity of that extremity (Wolf Motor Function Test, WMFT performance rate).** MAL outcomes at post-treatment and 1-year follow-up are graphed in **(A)** and **(B)**, respectively. Note that treatment outcome for spontaneous use of the more affected arm is increased 2.4 times by use of the TP. WMFT post-treatment outcomes are graphed in **(C)**. Note that shaping greatly increases maximum motor capacity made on request in the laboratory. In all three panels, change from pre-treatment is plotted. Reprinted from Taub et al. (2013b).

**FIGURE 3:**
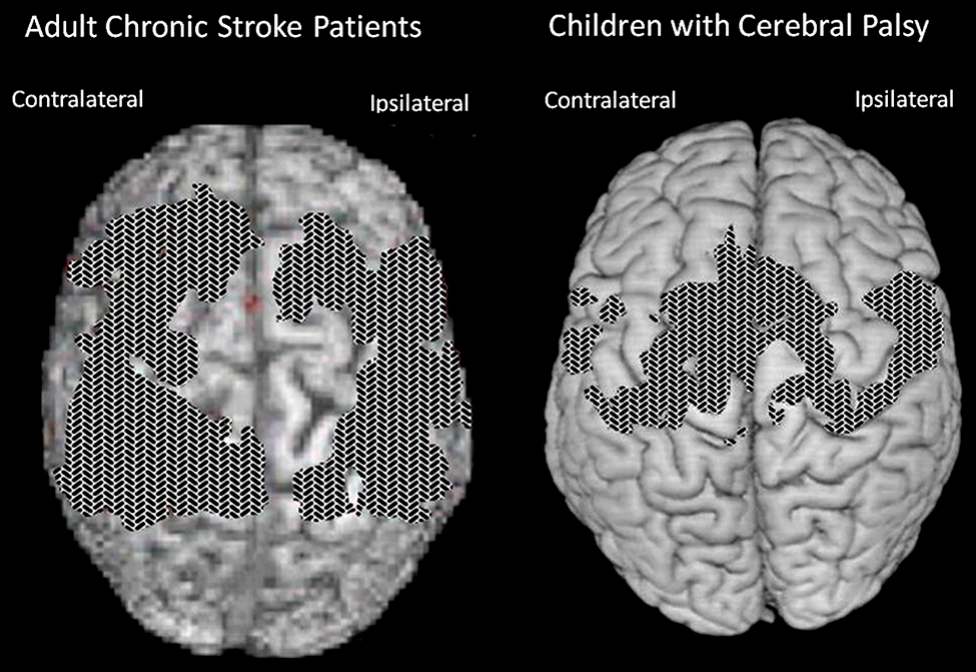
**Cortical surface-rendered image of gray matter pre- to post-treatment change after CI therapy in **(A)** adults with chronic stroke and **(B)** children with hemiparetic cerebral palsy.** Gray matter increases displayed on a standard brain. Surface rendering was performed with a depth of 20 mm. Cross-hatched areas indicate t statistics ranging from 2.0 to 6.7, corrected for family-wise error. Panel **(A)** adapted from Gauthier et al. (2008); panel **(B)** adapted from Sterling et al. (2013).

Use of the TP, regardless of the type of training received, resulted in MAL gains that were 2.4 times as large as the gains in its absence (*P* < 0.01; **Figure 2**). The MAL gains were retained without loss 1 year post-treatment. An additional substudy (*N* = 10) showed that a single component of the TP, weekly telephone contact with participants for 1 month after treatment, doubled MAL scores at 6-month follow-up, closing half the quantitative gap in real-world spontaneous movement between the two groups. Thus, the TP would appear to be a method for strongly enhancing spontaneous use of a more-affected arm in the life situation. After treatment, voxel based morphometry (VBM) analysis of MRI scans indicated that the two TP groups also exhibited a profuse increase in gray matter in the sensorimotor cortices, more anterior motor areas, and the hippocampus in both hemispheres (**Figure 3A**; Gauthier et al., 2008). The groups not receiving the TP showed no change in amount of gray matter after treatment. Additional results from this experiment indicated that shaping improved maximal motor performance made on request in the WMFT, but while it also improved real world spontaneous use of the more-affected limb significantly, it did so only a fraction as much as when the TP was used in addition.

The question might arise as to whether the TP increases treatment effect by simply increasing the amount of practice of more-affected arm use, since more-affected arm use is strongly encouraged in life situation ADL as well as in the clinic. Alternatively, it is possible that the TP promotes integration of therapeutic gains achieved in the laboratory into real-world activities so that more-affected arm use becomes habitual. These two possibilities are not mutually exclusive. Addressing this question in future research would be of mechanistic and theoretical interest; however from the point of view of practical therapeutics, the resolution of this important issue does not really matter. The TP appears to be a means of increasing real-world treatment outcome that does not involve increasing costly therapist time; this would be of considerable value whatever the mechanism by which the TP achieved its effect.

### CI THERAPY IN OTHER LABORATORIES

At UAB, over 1000 patients with stroke have been given one variant or another of CI therapy and all but four of these patients have demonstrated substantial improvement in motor ability (i.e., improvement greater than a minimum clinically important difference; Lum et al., 2004; van der Lee et al., 2004; Uswatte and Taub, 2005; Lang et al., 2008). There have also been approximately 600 papers from other laboratories on adult and pediatric CI therapy published to date. To our knowledge all but two of the studies have reported positive results. In particular, CI therapy was the subject of a multi-site RCT (Wolf et al., 2006); the results were strongly positive.

Some of the papers on CI therapy from elsewhere report outcomes as large as those obtained in this and other laboratories; however, most of these studies report results that are significant, but only one third to one half as large as those obtained here. The likely reasons for the reduced treatment effect in these laboratories are twofold: (1) there was incomplete or complete lack of use of the procedures of the TP, which, though reported in the papers from this laboratory, had been largely ignored. As noted above, we have replicated the reduced treatment effect obtained by others by duplicating everything that is normally done in treatment here except implementation of the TP (Gauthier et al., 2008; Taub et al., 2013b). (2) Another probable reason for obtaining reduced effects is that a protocol with attenuated intensity (tasks or movements per unit time) was used, such as in a study by van der Lee et al. (1999).

This laboratory’s results have been replicated with patients with chronic stroke in published studies from four laboratories where therapists were trained at UAB: the laboratories of Miltner and Bauder (Miltner et al., 1999), Flor and Kunkel (Kunkel et al., 1999), Elbert and Sterr (Sterr et al., 2002), and Dettmers and Weiler (Dettmers et al., 2005); in the first three studies, CI therapy was set up with the collaboration of one of us (E.T.) and then monitored twice yearly. In all four of these studies some but not all elements of the TP were employed. However, in each case attention was focused on the transfer of therapeutic gains in the laboratory to spontaneous use of the more-affected arm in the life situation and some TP elements were used.

The reason for going into this detail in describing the TP and the range of results obtained in other laboratories with what is designated by those authors as CI therapy, is that the same issues probably pertain to the treatment of sensory deficits by training as they do to CI movement therapy. The same consideration applies to some of the sections that follow.

### APPLICATIONS OF CI THERAPY

#### Severity and chronicity of motor deficit

Most of the patients treated at UAB could be characterized as having deficits that were mild/moderate. Experiments have also been carried out with patients with moderate and moderately severe deficits (grades 3 and 4; Taub et al., 1999). Their treatment change was somewhat less than for higher functioning patients, e.g., increases of approximately 400% and 350% for patients with moderate and moderately severe deficits, respectively, compared to approximately 500% for patients with mild/moderate deficits, but the treatment changes were nevertheless very large. Recently, work has been carried out with patients with useless, plegic hands that were initially fisted (Uswatte et al., 2008; Taub et al., 2013a). At the end of treatment, the patients exhibited a 186% improvement in the real-world use of the more-affected arm. It had been converted into a useful “helper” in the life situation. We estimate that CI therapy is applicable to at least 50% of the chronic stroke population with motor deficit, perhaps more.

There does not appear to be any upper limit to the length of time since stroke and the benefit that is obtained from CI therapy. There is also no correlation between chronicity and magnitude of treatment effect. The patient with the longest time period between the event and treatment was 50 years; stroke occurred at 5 years of age and treatment was given at 55. The improvement in more-affected arm movement was in the middle of this laboratory’s range. There is also no correlation between age and treatment outcome. Several patients have been treated in their 90s, and each one had a substantial improvement in motor function.

#### Lower extremity

CI therapy techniques have been applied to the more-affected lower extremity of stroke patients (Taub et al., 1999). The treatment (Lower Extremity-CI therapy or LE-CI therapy) consists of massed or repetitive practice of lower extremity tasks (e.g., over-ground walking, treadmill walking with and without a partial body weight support harness, sit-to-stand, lie-to-sit, stair climbing, walking over obstacles, various balance and support exercises). Task performance is shaped as in the upper extremity protocol. No restraining device is placed on the less-affected leg. The lower-extremity procedure is considered to be a form of CI therapy because of the use of the TP, the strong massed practice/shaping element, and because the reward of adaptive patterns of ambulation over maladaptive patterns in our training procedure constitutes a significant general form of constraint. This is another form of CI therapy where constraint is imposed by the training paradigms but there is no physical restraint.

#### Retention

The treatment effect is long-lasting. For the upper extremity, its persistence is related to the severity of the initial impairment. For patients with mild/moderate initial impairment – grade 2 (but who entered treatment because of markedly reduced use of the more-affected arm in the real-world environment; i.e., MAL score <2.5), retention at 1 year after treatment varied in different experiments around the 90% level; at 2 years retention was approximately 80%. For patients with moderate initial impairment (grade 3), retention at 1 year was 75%; for patients with moderately severe impairment (grade 4), retention after 1 year was 60%; and for patients with severe deficit (grade 5) with initially plegic hands, retention across two experiments was 46%. The situation for the legs is quite different. Retention after 1 year is approximately 100% for all grades of motor deficit studied, from mild/moderate to patients who are minimally ambulatory. Some patients do exhibit a decrement in performance at 1 year, but others continue to improve above their immediate post-treatment level. One might speculate that to ambulate and for most lower extremity functions, both legs must be used, the more-impaired as well as the less-impaired extremity. Thus, use of the more-affected leg is maintained, and retention of the treatment effect is high. For the arms, however, just one extremity can be used for many tasks, and since use of the more-impaired arm remains effortful, a part of the conditions that initially gave rise to learned nonuse remain in effect; thus, reduced use of the more-affected extremity gradually returns. The greater the effort to use the more-affected arm (i.e., the greater the impairment from grade 2 through grade 5), the greater the reduction in real-world arm use over time; so that a smaller amount of the treatment effect is retained. The same retention results as for the upper extremities with respect to retention of treatment effect are likely to apply to visual deficit training.

#### The broad range of applications to other motor disorders following CNS injury

**FIGURE 4:**
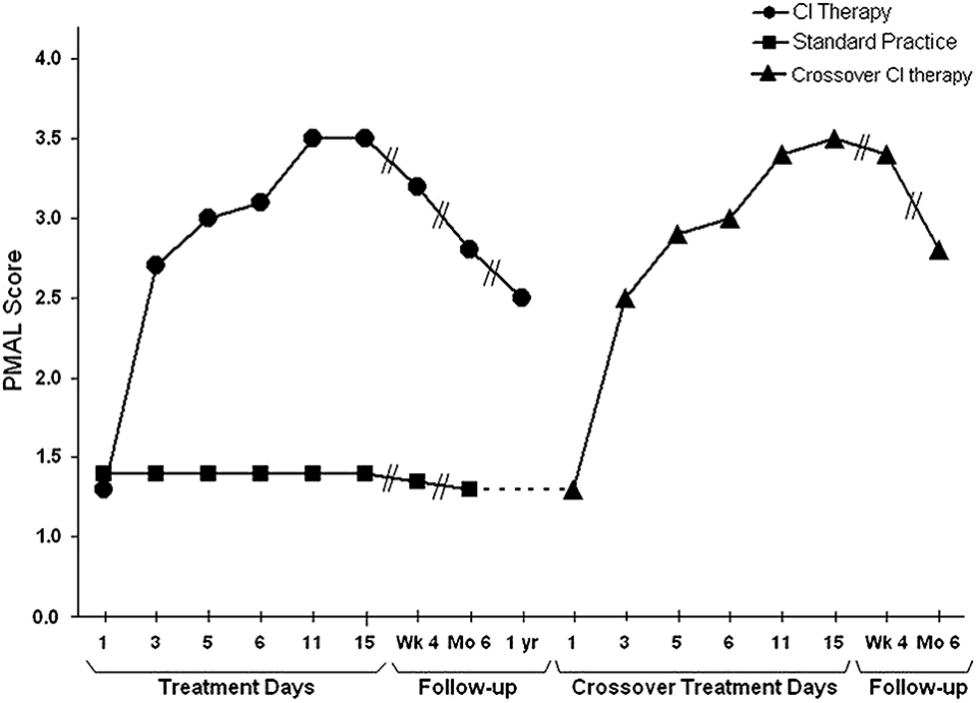
**Spontaneous use of the more impaired arm (Pediatric Motor Activity Log score) of young (2–6 years) children with hemiparetic CP receiving Constraint-Induced Movement therapy or standard occupational therapy.** Data for the Constraint-Induced Movement therapy group are shown before and during treatment and 1, 6, and 12 months after treatment. Data for the control subjects are shown at corresponding times for 6 months after treatment, at which time they were crossed over to Constraint-Induced Movement therapy. After crossover data are shown for treatment and 1 and 6 months afterward. The data are similar to those for adults shown in **Figure 1**. For both the children given CI therapy first and those given the intervention after crossover, the amount of spontaneous use of the more affected arm in the ADL in the real-world environment increased from approximately 15% compared to use of the less affected arm to approximately 65%. Reprinted from Taub et al. (2011).

The finding that after unilateral forelimb deafferentation in monkeys, training of the deafferented limb or restraint of the intact limb enabled extensive purposive movement in a previously useless upper extremity undermined the reflexological interpretation of movement. The need to explain the initial nonuse of the deafferented limb even though motor pathways were still intact, and the way this nonuse could later be overcome gave rise to the learned nonuse formulation. This formulation is predicated on the fact that any substantial damage to the CNS generally results in reduced excitability of neural structures with extensive connections to the directly injured area. This loss of excitability in turn results in a deficit in or complete loss of the function that the defacilitated neural tissue had previously supported. However, levels of excitability slowly recover spontaneously (Swayne et al., 2008; Takechi et al., 2014), and this is reflected in the process of spontaneous recovery of function. While monkeys with deafferented forelimbs were never observed to spontaneously recover the ability to use the affected forelimb purposively, the two techniques already described enabled extensive use of the deafferented extremity. Though these two techniques had to be applied for a period of days in the case of restraint of the intact limb or over multiple sessions with training for purposive movement to become long-term, the fact that purposive movement could be evoked in very short periods of time, hours in the case of physical restraint and several 1-h sessions in the case of training, suggested that the excitability of neural tissues had been restored previous to these interventions, after the initial period of reduced CNS excitability had dissipated. Why then was there no return of the motor function that should have been possible? To account for this situation, the learned nonuse formulation was developed (Taub, 1977, 1980; Taub et al., 2006b). However, though developed to explain this specific set of experimental facts, learned nonuse was viewed as a general phenomenon independent of specific neural structures. It would not matter from the point of view of the formulation, if a motor deficit were due to the abolition of an extremity’s afferent supply, or to a lesion in any of a number of different locations in the brain or spinal cord. These would presumably include lesions in: motor cortex, descending tracts in the spinal cord, basal ganglia, sensorimotor cortex, or more anterior motor areas. For that matter, injury could be to skeletal or other tissue, as long as there is a change in the organism that makes attempted use of a function punishing. Learned nonuse was thus considered to be the basis of what was termed “excess motor disability,” or disability in excess of what appears to be warranted by the organic damage sustained, a frequently observed phenomenon whose mechanism had been unexplained. According to the formulation, the initial reduction in neural excitability and loss of function was said to set up the conditions for the development of learned nonuse, which could convert the temporary early deficit in function produced by the initial neural defacilitation into a permanent condition unless appropriate techniques were applied. For the extremities the appropriate techniques for lifting the learned nonuse would presumably be the same whatever the location of the precipitating lesion. Thus, the learned nonuse formulation predicted that the approach employed with deafferented monkeys would also be applicable to humans after any of the above types of neural damage and to many different pathological conditions. The work described above showed that this prediction was amply confirmed for stroke. Given this promising start, an attempt was made to apply the general procedure that had been employed to motor deficits produced by other types of CNS damage and other diagnostic categories. On the basis of this formulation, these applications of CI therapy were straightforward. This prediction has been confirmed to date by the application of the CI therapy protocol with the same positive results to the upper extremity after TBI (Shaw et al., 2003), MS (Mark et al., 2008), cerebral palsy, and other pediatric motor disorders of neurological origin across the full range of age from 1 year old through adolescence (Taub et al., 2004, 2007, 2011; see **Figure 4**).

The pediatric motor deficits treated included those resulting from TBI, brachial plexus injury, congenital brain malformations, and hemispherectomy (Taub et al., 2009). An adaptation of CI therapy for the lower extremity has been carried out in adults not only after stroke, but also after spinal cord injury and fractured hip (Taub et al., 1999) and MS (Mark et al., 2013). Both in our adult and pediatric clinics we have also worked with numerous cases of brain resection and obtained results comparable to those with stroke when the initial motor deficits were similar (Taub et al., 2007).

### CI THERAPY AND NEUROPLASTIC CHANGE

**FIGURE 5:**
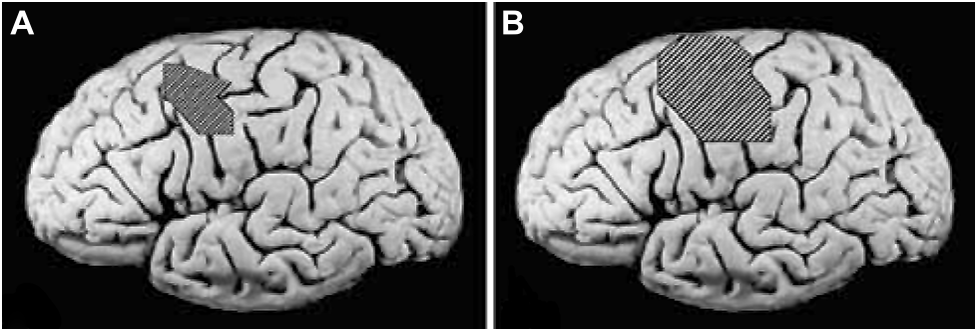
**(A)** Pretreatment cortical map of the excitable area for the contralateral abductor pollicis brevis in a group of stroke patients determined by transcranial magnetic stimulation (TMS), superimposed on an unlesioned post mortem brain to indicate approximate size and location. **(B)** Post-treatment TMS map in the same group of patients. Reprinted from Mark et al. (2006).

It has been found that CI therapy-type interventions involving training of extremity use after a CNS injury results in both improved extremity function and reorganization of brain activity. Nudo and co-workers demonstrated this first in new world monkeys (Nudo et al., 1996), showing that the area surrounding a motor cortex infarct that would not normally be involved in control of the hand came to participate in that function at the same time that performance on an experimental task involving manual dexterity improved. In humans whose upper extremity function had been enhanced by CI therapy, Liepert, Taub, and co-workers (Liepert et al., 1998, 2000b) used focal transcranial magnetic stimulation to show that the cortical representation of an important muscle of the hand (abductor pollicis brevis) was greatly enlarged. The results are presented in **Figure 5**.

**FIGURE 6:**
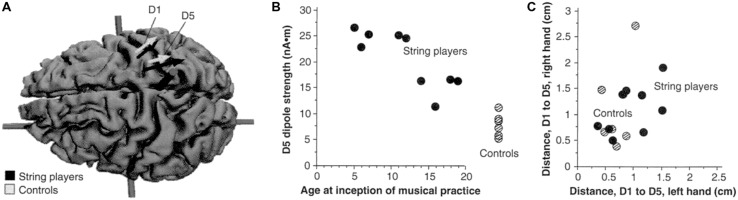
**(A)** Equivalent current dipoles elicited by stimulation of the thumb (D1) and fifth finger (D5) of the left hand are superimposed onto an magnetic resonance imaging (MRI) reconstruction of the cerebral cortex of a control, who was selected to provide anatomical landmarks for the interpretation of the MEG-based localization. The arrows represent the location and orientation of the ECD vector for each of the two digits’ averaged across musicians (black) and controls (shaded). The length of the arrows represents the mean magnitude of the dipole moment for the two digits in each group. The average locations of D5 and Dl are shifted medially for the string players compared to controls; the shift is larger for D5 than for Dl. The dipole moment is also larger for the musicians’ D5, as indicated by the greater magnitude of the upper arrow. **(B)** The magnitude of the dipole moment as a function of the age of inception of musical practice; string players are indicated by filled circles, control subjects by hatched circles. Note the larger dipole moment for individuals beginning musical practice before the age of 12. **(C)** Scatterplot of the Euclidean distances (in centimeters) between the cortical representations of Dl and D5. This distance for the musicians’ left hands was greater than that in controls, but this difference is not statistically significant. Reprinted from Elbert et al. (1995).

The finding that CI therapy is associated with substantial changes in brain activity was confirmed in other early studies in which one of us (E.T.) also collaborated involving the Bereitschafts potential (Bauder et al., 1999), positron emission tomography (Wittenberg et al., 2003), and EEG source-imaging (Kopp et al., 1999). To date, there have been more than 20 studies, many involving functional magnetic resonance imaging, that have obtained similar results (summarized until 2006 by Mark et al., 2006). These studies employed functional brain imaging and brain mapping techniques. The question remained whether CI therapy could measurably alter brain structure in humans. Starting approximately 15 years ago it was shown that increased use of a function or body part could result in an increase in the amount of regional gray matter in the part of the brain associated with that function. For example, it was shown that the cortical representations of the left hand of string players were larger than those of control subjects (see **Figure 6**). It was also found that experienced taxi drivers have significantly expanded hippocampi (Maguire et al., 2000), and jugglers acquire significantly increased temporal lobe gray matter density (Draganski et al., 2004), among many other examples. Conversely, thalamic gray matter density significantly declines after limb amputation (Draganski et al., 2006). Moreover, in an animal model of stroke, CI therapy combined with other exercise reduced tissue loss associated with stroke (DeBow et al., 2003). Thus, it became appropriate to ask whether there are anatomical changes following the administration of CI therapy and whether these are correlated with clinical improvements.

Longitudinal VBM was performed on subjects enrolled in our study of the contribution made by the TP to CI therapy outcome (Gauthier et al., 2008). It was found that structural brain changes paralleled changes in amount of use of the impaired extremity for ADL. Groups receiving the TP showed profuse increases in gray matter tissue in sensorimotor cortices and more anterior motor areas both contralateral and ipsilateral to the more-affected arm, as well as in bilateral hippocampi (see **Figure 3A**). The increases in gray matter were significantly correlated with increases on the MAL for the sensorimotor clusters on both sides of the brain and the predefined hippocampus region of interest (*r*s > 0.45). Thus, this change in the brain’s morphology is directly related to administration of the TP which in turn substantially increases the amount of real-world use of the affected arm. In contrast, the groups that did not receive the TP showed significant but relatively small improvements in real-world arm use and failed to demonstrate gray matter increases. The fact that the anatomical change is directly related to the TP lends increased credibility to the importance of the TP.

In another study (Sterling et al., 2013), children with hemiparetic cerebral palsy also showed increases in gray matter in the bilateral sensorimotor cortices (see **Figure 3B**). These changes showed a strong correlation with improvements in spontaneous real-world arm use as recorded on the pediatric version of the MAL. In more recent work, patients with progressive MS were found to benefit from CI therapy to the same extent as patients with stroke. In addition they also exhibited a substantial increase in gray matter in sensorimotor areas of the brain (Mark et al., 2014).

### APPLICATION OF CI THERAPY TO NON-PARALYTIC DISORDERS

#### Speech

In the context of the present article in which the extension of the CI Movement therapy protocol to visual deficits is considered, an application of CI therapy of particular interest is its use with post-stroke aphasia, since this involves a non-motor deficit. In a substantial number of stroke patients, because of halting and slow verbal production and incomplete understanding, speech becomes very effortful and often embarrassing. The person compensates by greatly reducing attempts to speak or remaining silent entirely and by using gestures and other non-verbal means of communication. The demonstrations described above that learned nonuse associated with motor deficits is modifiable in chronic stroke raised the possibility that verbal impairment could also be rehabilitated by an appropriate modification of the CI therapy protocol. The LNU formulation predicted that this was a strong possibility. In the initial study, by Pulvermüller, Taub, and coworkers (Pulvermüller et al., 2001; Taub, 2002), aphasic patients with chronic stroke who had previously received extensive conventional speech therapy and had reached an apparent maximum in recovery of language were induced to talk and improve their verbal skills by engaging them in a card game that required frequent and detailed spoken requests and replies for 3 h each weekday over a 2-week period. The intervention was termed Constraint-Induced Aphasia therapy (CIAT I). Constraint was imposed by the requirements of the training/shaping paradigm that was used; there was no physical restraint, though as noted, physical restraint is not necessary to obtain a good result with CI Movement therapy for the upper extremity and it is not used at all in this laboratory for the lower extremity. This study has since been replicated (e.g., Bhogal et al., 2003; Meinzer et al., 2004, 2007; Maher et al., 2006; Kirmess and Maher, 2010). While the results of the CIAT I protocol have been positive, the intervention was only an incomplete translation of CI Movement therapy. CIMT produces an improvement of approximately 500% in real-world use of the more-affected extremity of chronic stroke patients with mild to moderate motor deficit in the UAB laboratory (Taub et al., 2006a). Aphasic patients given CIAT I improved by 30% in real-world verbal behavior. This is a large treatment effect compared to conventional speech language therapies, but it is very small compared to the results produced by CIMT. Consequently, to determine whether the large difference resulted from an incomplete translation of the CI therapy protocol employed in the UAB laboratory with motor deficits to the treatment of language impairment, the initial aphasia treatment protocol (CIAT I) was modified to more closely resemble the CIMT protocol.

**FIGURE 7:**
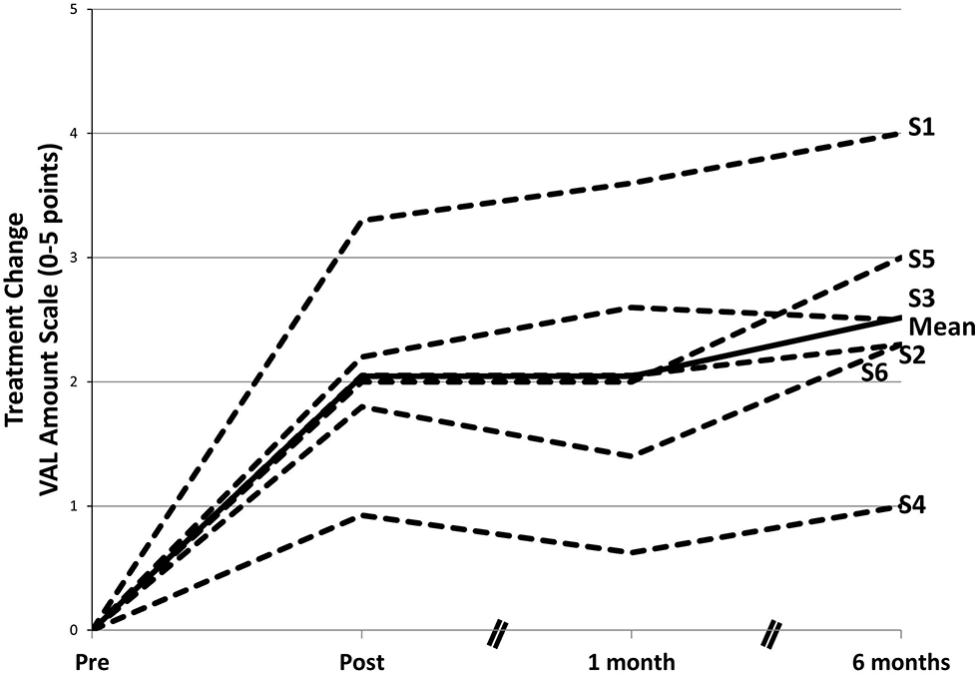
**Changes in amount of participation in speech in life situations after a course of CIAT II.** The mean gain from pretreatment on the Verbal Activity Log (VAL) amount of use scale was significant at posttreatment. After treatment, there was an additional significant performance gain. Data in the graph are ipsitized—that is, pretreatment scores are set to 0 for each participant, and subsequent scores are reported as changes from pretreatment. S1–S6 refer to subject numbers. Modified from Johnson et al. (2014).

In the restructured and enhanced protocol (CIAT II; Johnson et al., 2014), revisions involved addition of new exercises, including a final exercise, considered to be the most important, in which everyday verbal interactions were simulated and modeled. In addition, a TP parallel to that used in CIMT was introduced, there was increased emphasis on the shaping of responses, and the primary caregiver was trained as an alternate therapist with their training beginning in the laboratory but focused largely on the at-home practice of verbal behavior. To date, six patients have been treated with the new protocol (four reported in Johnson et al., 2014). Their results have far exceeded those obtained with CIAT I and are more comparable to the results obtained with CIMT. With CIAT I, as noted, there was a 30% improvement in real-world verbal behavior; for the recent patients, the mean improvement was approximately 300%. Of additional interest is the fact that in the 6 months following the completion of treatment verbal behavior scores increased substantially (see **Figure 7**). This increase would appear to be attributable to the continuation of training by the caregivers in the real-world environment and other aspects of the TP.

#### Focal hand dystonia

Focal hand dystonia involves manual incoordination that occurs in individuals, including musicians, who engage in extensive and forceful use of the digits. It involves loss of ability to make use of one or more digits independent of movement of another digit. Using magnetic source imaging, we found that musicians with focal hand dystonia exhibit a use-dependent overlap or smearing of the representational zones of the digits of the dystonic hand in the somatosensory cortex (Elbert et al., 1998, 2000). M. Hallett’s laboratory obtained similar results (Bara-Jimenez et al., 1998).

From the vantage of this article, this application of CI therapy is of interest because it was used to treat a deficit that was not produced by destruction of CNS tissue, and though the deficit treated was motor in nature, it involved disordered cortical representation zones in a sensory area of the brain. Digital overuse had previously been found to produce a similar phenomenon in monkeys in the laboratory of M. Merzenich. Since behavioral mechanisms apparently underlie both the cortical disorder and the involuntary incoordination of movement, we hypothesized that a behavioral intervention could reduce or eliminate both of these correlated abnormalities. Learned nonuse was not hypothesized to be involved in the disorder, but it will be remembered that CI therapy when used to treat patients with stroke, TBI, MS, and other conditions not only overcame learned nonuse, but produced a large improvement in impairment and movements in the laboratory when participants were requested to perform at a maximal level.

Eight professional musicians (six pianists and two guitarists) with long-standing symptoms were studied (Candia et al., 1999, 2002). Our therapy involved immobilization by splint(s) of one or more of the digits other than the focal dystonic finger. The musicians were required to carry out repetitive exercises with the focal dystonic finger in coordination with one or more of the other digits for 1.5–2.5 h daily (depending on patient fatigue) over a period of eight consecutive days (14 days in one case) under therapist supervision. The practice was thus massed; practice of this intensity and duration was very taxing and was at the limit of the patients’ capacity. The movements in the laboratory were continuously shaped, the patients were given daily home practice exercises, and a derivative of the MAL was administered each morning before therapy in which problem solving was carried out. After the end of an initial period of treatment, the patients continued practicing the exercises with the splint for 1 h every day or every other day at home in combination with progressively longer periods of repertoire practice without the splint.

All patients showed significant and substantial improvements without the splint at the end of treatment in the smoothness of finger movement, as determined by a device that measured finger displacement, and self-reported dystonia symptoms. The improvement persisted for the 2 years of follow-up in all the patients but one who did not comply with the home practice regimen prescribed. Half of the subjects returned to the normal or almost normal range of digit function in music performance. The treatment is characterized as a form of CI therapy because it has all its main components: massed practice, the main elements of the TP, frequent feedback during exercises, shaping of improved finger movements, and restraint of a body part.

As noted above, focal hand dystonia does not appear to involve learned nonuse. That is, there does not seem to be an advantage in not being able to make independent use of the digits, nor is there any apparent period of CNS defacilitation when an inappropriate pattern of coordination could be learned and “locked in” by being partially successful. Nevertheless, a CI therapy-approach improved function. This may be related to the fact, discussed above, that CI therapy not only overcomes learned nonuse, thereby substantially improving function in the life situation, but also improves impairment. This becomes relevant when considering the potential application of a CI therapy approach to the rehabilitation of visual and other sensory systems after CNS damage.

#### Amblyopia

In a previous article (Taub, 2010) we have noted the striking similarities in the procedures and magnitude of treatment results between CI therapy and the type of amblyopia treatment developed by Polat, Levi, and their respective co-workers (summarized in Levi and Polat, 1996). The parallels between the two treatments are reviewed here to establish the possibility that the CI therapy method might produce similar results with other types of visual impairments.

Levi and Polat treat amblyopia by intensive training while occluding the non-amblyopic eye during training. The treatment effects reported are very large. In both amblyopia training and CI movement therapy for the upper extremity the unimpaired part of the body is not permitted to participate in accomplishing the training task, in amblyopia training by patching the sound eye during the training period and in CI therapy by use of a restraint device or sometimes more simply by having the therapist verbally discourage use of the less affected arm during the training period (Uswatte et al., 2006a). Similarly in CI Aphasia therapy non-verbal modes of communication are strongly discouraged, thereby imposing a strong constraint on behavior; physical restraint is not used. Training in each of these types of treatment starts with easily accomplished tasks at or just a little beyond present ability, and after mastery is demonstrated, the individual is challenged with more difficult tasks. In behavioral psychology this process, as noted above, is termed shaping, in which tasks are made more difficult progressively, typically in small steps (Skinner, 1938, 1968; Taub et al., 1994). In both amblyopia training and all forms of CI therapy with adults, immediate trial-by-trial feedback of results is provided and in all cases this is felt to be an important factor in patient improvement. In addition, in the work of Levi and Polat, the nature of the discrimination tasks employed in the training is tailored to the specific deficits of individual patients.

Both CI therapy and amblyopia training rely heavily on generalization of improved ability from the trained task to other functions. The basic measure for efficacy in amblyopia is improvement in visual acuity as measured in standard fashion by Snellen charts at a 3 m distance. In different experiments by Polat, Levi and others with adult amblyopes, the training tasks employed have included a contrast sensitivity task with flankers (e.g., Polat et al., 2004; Zhou et al., 2006; Huang et al., 2008), vernier acuity, and position discrimination (Levi and Li, 2009). Despite evidence of specificity (e.g., no improvement at untrained orientations), the procedures in all experiments led to improvement in Snellen acuity. Moreover, other degraded visual functions which were not trained nevertheless improved substantially, such as stereoacuity and visual counting (Li and Levi, 2004; Li et al., 2007). Similarly, the training in the laboratory/clinic carried out in CI therapy which includes few full ADL, nevertheless transfers very extensively to the life situation, as measured by the MAL which, as noted, obtains information about 30 ADL important in life functioning, in such areas as eating, grooming, and dressing (Taub et al., 1993, 1999; Uswatte et al., 2000, 2005a,b, 2006b), and confirmed by objective accelerometry (Uswatte et al., 2000, 2005a,b, 2006b).

A recent advance in the treatment of amblyopia has come from training binocular viewing rather than forced use of the amblyopic eye by patching the good eye (Li et al., 2013; Mansouri et al., 2014). Patients are trained on a computer game, with the contrast levels for each eye adjusted to improve stimulus detection by the weaker eye, and requiring binocular viewing to score points. Preliminary findings indicate greater improvement in visual acuity with dichoptic stimulation over monocular patching, along with improved stereopsis. This result is not dissimilar from a form of rehabilitation termed bimanual training that has recently emerged in the stroke motor rehabilitation field for which good results have been reported. However, since the therapy involves using tasks that can only be accomplished effectively by using both arms, the participant is constrained to use the more-affected arm to carry out the task, this may be viewed as a form of CI Movement therapy, which may explain its relative efficacy. The same result from visual deficit training is achieved in the recent papers involving training with binocular viewing by adjusting the contrast levels for the two eyes so that the participant was constrained to place greater reliance on the amblyopic eye than under normal viewing conditions. Normalized visual acuity has not so far been obtained with binocular training (Li et al., 2013). Incorporating CI therapy methods with binocular training could potentially produce improved results and should be attempted.

In summary, both CI therapy and amblyopia training prominently use constraints on function which force or increase motivation to use the impaired portion of the body and set up the conditions for positive reinforcement when this results in improvements, however small, in the target function. The constraints include physical restraints: a padded mitt which prevents use of the less affected hand after damage to the nervous system in CI therapy and either patching the sound eye or equalizing stimulus strengths between the eyes by adjusting contrast levels during amblyopia training. It also involves more general methods for inducing use of the impaired body part. For example, in both types of treatment, training especially by shaping requires an individual to keep improving performance with the affected portion of the body. This is viewed as a constraint on behavior rather than a restraint, but it has the same effect. (For the most recent discussion of the role of constraints in CI therapy, see Taub et al., 2006b; Taub, 2012).

In one study using the amblyopia training procedure, adults whose defect was not too severe were trained in half-hour sessions given four to six times a week (Polat, 2008). Asymptote in treatment effect was often reached in 30–40 sessions, or 15–20 h of treatment (Levi and Li, 2009). In contrast, occlusion-only treatment usually continues for 100–400 h (Cleary, 2000; Stewart et al., 2004). The situation is very similar for CI therapy. An early attempt at applying the CI therapy procedure with adults (Wolf et al., 1989) involved use of just one-half of the suggested protocol (Taub, 1980). The less affected arm of chronic stroke patients with mild/moderate motor deficit was restrained in a sling for 90% of waking hours, but no training of the more affected arm was employed. Treatment outcome was measured on a laboratory motor function test (WMFT). The results were reliable, but small (ES *d′* = 0.2). Similar results were obtained in another laboratory (Ploughman and Corbett, 2004). However, when training of the more affected arm was added to the regimen, the effect recorded on the WMFT was four times as great (Taub et al., 1993, 2006a); the ES was 0.8–1.0. (As was noted above, the ES for real-world arm use as measured by the MAL was greater still.) Thus, as in the case of amblyopia treatment when only restriction of the unimpaired eye is used without training, the outcome is very much reduced. Training the affected part of the body greatly enhances the treatment effect.

The greatest gains in amblyopia training occur within the first eight sessions; the rate of improvement then slows and asymptote is reached in a mean of 30–40 0.5 h sessions (Li et al., 2007). This describes the negatively accelerated curve typical of many learning situations. The same pattern is exhibited in CI therapy for the upper extremity with both children (Taub et al., 2004) and adults (Taub et al., 1993, 2006a).

The mean visual acuity in the amblyopic eye before the beginning of training in one study was 20/70 to 20/80 (Levi and Li, 2009). At the end of a mean of 35 sessions (17.5 h) when the average patient reached an asymptote, Snellen acuity typically ranged from 20/20 to 20/40; that is, normal or very nearly. This treatment effect was certainly dramatic, but the initial deficit was only mild or at most moderate. Data from several hundred adult CI therapy participants indicate that patients with initial grade 2 (mild/moderate) motor deficit start treatment using their more affected arm spontaneously in the life situation approximately 9% of the amount they used it before stroke. After 2 weeks of treatment that amount increases to a mean of 52% (e.g., Taub et al., 2006a). This is an approximate five times increase, but it is not by any means a “cure.” Patients are still using their more affected upper extremity spontaneously in the life situation only half as much as they did before stroke. With lower functioning patients, the treatment change (as contrasted with the absolute level of function observed) is not quite as great, but it is similar. Severe amblyopes require approximately 50 h of training to reach a performance asymptote. There can be as much as a fivefold improvement over the course of treatment. The absolute level reached, though, can frequently be very much less than in the studies with participants with more moderate deficit (Li and Levi, 2007; Li et al., 2007). More generally, it is difficult to compare magnitude of treatment effects across such very different domains as visual acuity and motor deficit in the extremities. It may be that the improvement in visual acuity resulting from the perceptual training protocol in amblyopia is somewhat greater than for extremity movement in stroke or TBI patients following CI therapy. However, this is by no means certain, and an attempt at detailed comparison may not be profitable at this early stage. The clearest conclusion that can be reached is that very large improvements in function can be produced by use of the appropriate technique in both amblyopia and extremity motor deficit after stroke or brain damage resulting from a variety of causes.

In one study improvement in position discrimination remained stable after monocular training for the 3–12 months tested (Li and Levi, 2004). In other studies, a high level of retention is reported for monocular training 12 months after treatment (Polat et al., 2004; Zhou et al., 2006). For CI therapy, we have observed a clear, linear relationship between persistence of the treatment effect and severity of initial deficit for grade 2 (mild/moderate deficit) to grade 4 (moderately severe deficit) patients. For grade 2 patients at the end of 1 year there is an approximate 15% decrease at the end of 1 year. For lower functioning patients retention is less (Taub et al., 1999). However, in the EXCITE multi-site RCT, the retention in upper extremity function was 100% for grade 2 and 3 patients (Wolf et al., 2008). In our laboratory for lower extremity CI therapy retention is greater than for the upper extremity. Similarly, at 1 year after CI Aphasia therapy II (CIAT II) there was not only no loss in retention, but verbal ability improved significantly (19%). The improvement following the end of treatment is probably due to a strong emphasis on TP procedures (Johnson et al., 2014).

Prior to the work on amblyopia training, the treatment of amblyopia was not generally undertaken after the age of 9 and rarely beyond adolescence (Levi and Li, 2009). Though there were scattered, largely anecdotal, reports of success with sound–eye occlusion in adults, these were largely ignored. The assumption was that once the “critical period” for visual development had been passed, or was too long in the past, the potential for major modification of visual acuity was no longer possible. The amblyopia training studies just noted have unequivocally demonstrated the incorrectness of this traditional, almost axiomatic, belief. This is a major finding. Polat (2008) reports that among 44 patients ranging in age from 9 to 55 years the correlation between amount of improvement and age was not significant. The important comparison of the treatment effect for amblyopia training for children younger than 9, where traditional occlusion-only therapy has been concentrated, and persons in older age ranges has apparently not been made. Further research in this area would be of value.

In parallel research with CI therapy, the initial studies showed that this intervention produces a large and reliable improvement in motor function of the more affected arm of adult chronic stroke patients. More recent work shows that individuals from 18 months to 92 years who have been treated with CI therapy show no correlation between age and amount of spontaneous use of the more affected arm in the life situation.

## REHABILITATION OF FUNCTION IN OTHER SENSORY SYSTEMS AFTER CNS DAMAGE

There has been substantial work on improving deficient sensory function by training after CNS damage and as a result of congenital disorders. This work has typically used a part or parts of a CI therapy-type approach but not all of it. The CI therapy protocol involves, as already noted: (1) massed practice, (2) shaping, (3) a TP of techniques to bridge the gap between treatment setting and life situation, and (4) restraint/constraint of compensatory dominance of a more-intact portion of the body. This work will be reviewed briefly here. The results have been generally positive. However, papers reporting results for each separate modality tend to have a narrow focus and there is little reference to similar rehabilitation or remediation research done in other modalities, to say nothing of results of research directed toward improving deficits in motor systems. The question that this article asks is whether the results to date could be increased by adapting a comprehensive CI therapy-type approach to these other types of deficits.

### SOMATIC SENSATION

Given that motor deficit can be substantially reduced after CNS injury by, for example, a procedure such as CI therapy, it might not be unexpected that the sensory system most intimately connected to movement, somatic sensation, would also be amenable to enhancement by training after CNS injury. As Sherrington famously noted, somatic sensation follows movement like a shadow (unless, of course, that intimate connection is artificially abrogated, as by the surgical section of all dorsal roots innervating a body part while leaving the motor outflow over the ventral roots intact). Moreover, a large subliterature amply demonstrates that the cortical somatosensory representations of different portions of the body sustain a high degree of neuroplastic change following the alteration of peripheral afferent inflow (summarized in Buonomano and Merzenich, 1998; Taub et al., 2014). The expectation that impaired somatic sensation after CNS damage can be improved by training is borne out by the experimental evidence. In the early literature Ruch et al. (1938) reported that training could improve sensory discrimination in primates and man after parietal lesions. Similar results were obtained in later work (Forster and Shields, 1959; Vinograd et al., 1962; Van Deusen Fox, 1964; De Jersey, 1979; Yekutiel and Guttman, 1993; Knecht et al., 2001). Though, as Smania et al. (2003) point out, flaws in individual studies prevent clear interpretation of the results in detail, the main finding that somatosensory deficits can be reduced after CNS damage by training emerges clearly. In one study of interest it was found that training in proprioception or position sense at the elbow improved discrimination in that submodality, but the improvement did not generalize to tactile sensation. When training was given in more than one somatosensory submodality, each of those sensory modalities showed improvement (Carey et al., 1993; Sormani et al., 2014). In a recent study with two patients with somatosensory deficit after stroke, multimodal somatosensory training was given for 4 h/day for 10 consecutive weekdays, a schedule of training similar to that employed in CI therapy. The participant with the more severe deficit showed no improvement, however, the participant with the lesser deficit significantly improved in tactile perception, proprioception, and blind match to samples of palpated novel objects (Borstad et al., 2013). These studies in general, then, demonstrate that impaired somatosensory function after stroke can be improved by training. However, none of these studies employed two of the important components of CI therapy; shaping to progressively increasing levels of performance proficiency and TP techniques. It is possible that the reported improvements in impaired somatosensory discrimination could have been improved by adding these procedures.

### HEARING

A forced use training approach to acute unilateral sensorineural hearing loss was recently attempted, inspired by CI movement therapy (Okamoto et al., 2014). Patients were randomized either to usual corticosteroids or to corticosteroids plus listening to classical music through a headphone with only the impaired ear for 6 h per day for 9–10 days, while the other ear’s auditory canal was plugged. The sound levels and frequency distributions were self-adjusted to approximate pre-illness experiences. The investigators observed that the experimental approach was associated with significantly better pure tone audiograms relative to steroid treatment alone and were maintained at 2–3 months follow-up. The study also found improved laterality indices of auditory evoked responses as measured by magnetoencephalography following the experimental intervention relative to baseline. Although a complete CI therapy approach was not used, the findings are encouraging and suggest that more of a CI therapy modification could further benefit treatment outcomes.

### VISION

#### Unilateral spatial neglect

Unilateral spatial neglect is a common consequence of stroke occurring in about half of all survivors in the acute phase. It refers to a failure to report, respond to, or orient to stimuli primarily in one part of space, most often opposite to the side of the brain injury (Mark, 2003; Barrett et al., 2012). It results in the disturbance of basic functional activities such as feeding, grooming, locomotion, orientation of the body in space, activities involving eye-hand coordination and other types of “motor aiming.” Neglect is not purely a disorder of visual input. It can be manifest in the dark (Karnath et al., 1998), in lingually clearing one’s own mouth (André et al., 2000), and when describing the spatial layout of distant familiar landmarks while in the laboratory (Guariglia et al., 2013). However, because it is most often observed during visual tasks, we will discuss it as a visual disturbance here. Although the condition is termed “neglect” because of underattention toward one direction, it can also manifest hyper-attention in the opposite direction (Mark et al., 1988), and thus should best be considered an imbalance in the spatial distribution of attention.

Unilateral neglect frequently resolves spontaneously during the first year after brain injury, yet many patients remain chronically impaired during daily living activities (Kinsella and Ford, 1985). Treatments for unilateral neglect have been largely unsuccessful or impractical, or they have not been evaluated in controlled studies (Mark, 2003). A therapeutic approach that is commonly used is termed visual scanning training (Lawson, 1962; Weinberg et al., 1977; Toglia, 1992; Quintana, 2002). Two strategies are to place a highly distinctive stimulus such as a colored stripe in contralateral space (“anchoring”) to overcome scanning biases, or teaching the patient to use their finger to guide themselves contralaterally. Some patients have been observed to benefit from this type of intervention. However, not all patients benefit, and systematic studies on the efficacy of this approach have not been conducted (Mark, 2003; Barrett et al., 2012). One intervention that has received considerable attention in the last decade is the use of adaptation to the lateral displacement of vision in the direction opposite to the neglected field of vision. Since most unilateral neglect occurs after a stroke affecting the right hemisphere leading to neglect of the left visual hemi-field, prismatic lenses that displace the visual field to the right are used in the therapeutic intervention. Initially the subject, including patients with left neglect, make errors in pointing to a visual target in a rightwards direction. If subjects are allowed to see their executive hand, pointing is rapidly corrected leftwards so that pointing becomes accurate. If the goggles containing the prisms are then removed, the subject makes an error in pointing, termed the aftereffect, in the opposite direction, that is leftwards. For patients with left neglect this in part corrects for part of their deficit, although transfer to real-world daily living activities has seldom been evaluated. The improvement has been found to persist in some patients (Barrett et al., 2012). For other patients the improvement is short-lived. In general the results have been mixed (Barrett et al., 2012).

An important parcellation of the unilateral spatial neglect phenomenon has been made between a visuo-motor aiming deficit and a disturbance in “spatial where” perception (Adair and Barrett, 2008; Eskes and Barrett, 2009; Heilman et al., 2011). The two undoubtedly have an overlap in brain regions in terms of the causative damage (Goedert et al., 2014). Empirically they are separate issues, and this is probably true also for methods of achieving therapeutic remediation. Goedert et al. (2012) using the Catherine Bergego scale (Azouvi et al., 2003) found differential perceptual where and motor aiming deficits. It has also been found that “visual where” perception does not improve after prism adaptation therapy while motor aiming behavior usually does (Striemer and Danckert, 2010; Goedert et al., 2014). Moreover, the opposite is also true. Some patients with apparently intact ability to perceive and represent the visual environment nevertheless make persistent motor aiming errors (Laplane and Degos, 1983; Coslett et al., 1990; Triggs et al., 1994; Barrett et al., 1999).

The dissociability of remediation of “visual where” perception and motor aiming deficits may be based on differences in their underlying corrective mechanisms. In the older literature there was an experimental debate on whether adaptation of pointing at visual targets to lateral displacement of vision by wedge prisms involved a recalibration of vision (e.g., Held and Hein, 1958; Held, 1965) or, perhaps counterintuitively, a recalibration of position sense of the executive arm (Harris, 1963, 1965; Hochberg, 1963; Pick et al., 1963; Hamilton, 1964). It appeared that a critical method for evaluating these positions would be to determine how the surgical abolition of the theoretically relevant proprioception from the executive arm would affect the ability to compensate for prismatic displacement of vision. Deafferentation was achieved by the serial section of dorsal roots from the second cervical to the third thoracic segment. Monkeys with a deafferented forelimb were first trained to point with reasonable accuracy at a visual target without view of the body or limbs, receiving feedback on the terminal position of the pointing finger only via food reward (Taub et al., 1975a). After adaptation to displacement of vision occurred, the helmets with prism lenses were removed and the course of the aftereffect was tracked (Taub and Goldberg, 1975). For normal animals the prism aftereffect was 39% of full prism displacement; for the deafferented animals, it was 100%. It thus seemed clear that the presence of proprioception inhibits adaptation to laterally displacing prisms.

Earlier research (Taub and Berman, 1963, 1968; Taub et al., 1973) presented evidence that position sense consists of two independent, redundant components: one peripheral (usually termed proprioception) and one central (variably termed central feedback loops or central efferent monitoring). In monkeys with a deafferented neck and forelimbs, the peripheral component is absent; there is thus less resistance to recalibration of the arm during prism adaptation and, accordingly the process proceeds to completion more rapidly and is more stable than in normal subjects. One might say that the less there is of position sense, the easier it is to recalibrate, and the more resistant to alteration the recalibration is (as evidenced by the larger aftereffect in deafferented monkeys). The recalibration of body parts can be rather specific. For example, in a visuo-motor pointing experiment, one normally gets a recalibration of the pointing extremity only with no intermanual transfer except under specially arranged circumstances (e.g., appropriate spacing of pointing practice trials; Taub and Goldberg, 1973). Thus, if prism adaptation involves a recalibration of position sense and not vision, this would explain why prism adaptation therapy for unilateral spatial neglect after stroke would correct the deficit in motor aiming, but have a reduced effect on the deficit in visual where perception.

This does not mean that vision cannot be recalibrated. For example, Taylor and Papert (1955) noted that some of their subjects who wore spectacles that inverted the visual field eventually reported after a period of days that the perceived world no longer appeared to be inverted. A similar phenomenon had previously been reported by (Stratton, 1897). There has been controversy as to whether Stratton did in fact experience an upright world (Carr, 1935) or whether he did not (Ewert, 1930, 1937; Woodworth, 1934; Higginson, 1937; Peterson and Peterson, 1938). Snyder and Pronko (1952) repeated Stratton’s experiment but could not give a conclusive answer to the question. There is clearly a verbal ambiguity in the question of whether the visual field appears upright. While this question and its analysis are of conceptual interest, in terms of practical import there is no question that humans can adapt to massive transformations of the visual field. The recalibration of vision (or perhaps the recalibration of direction of gaze) is probably responsible for the partial success in some patients of visual scanning therapy (Weinberg et al., 1977), training of visual awareness (Tham et al., 2001), therapist-coached use of mental imagery (Niemeier et al., 2001), and review with a therapist of videotaped feedback of task performance (Tham and Tegnér, 1997). The fact that the success of these techniques is limited is not surprising in view of the report of Taylor and Papert (1955) and other investigators (Stratton, 1897; Ewert, 1930; Kohler, 1951; Kottenhoff, 1957; Ardiago, 1886; as reported in Giannitrapani, 1958) that adaptation to displacement, reversal, and inversion of vision requires continuous experience with the transformed vision over a period of many days. The study by Antonucci et al. (1995) is relevant in this regard. These authors reported that comprehensive scanning training and prolonged practice with reading, copying, and describing pictures resulted in significant improvement on standard and functional aspects of unilateral neglect that had not been directly trained. The authors suggested that the basis for their successful behavioral intervention was “massive stimulation.” Training sessions were 1 h daily, 5 days a week, for eight consecutive weeks, or 40 h total. This is considerably more than is allotted for the therapies for unilateral post-stroke neglect noted above. This report is consistent with the extended experience required for adaptation to inversion and reversal of the visual field in healthy adults. It is also consistent with the amount of training that is given in CI therapy for motor deficit after stroke.

There are other parallels with some phenomena associated with unilateral neglect and one of the mechanisms that underlie a portion of the motor deficit after stroke that is improved by CI therapy. Neglect patients commonly fail to protect the paretic limb during transfer from bed to chair or during mat mobility (Mark, 2003). Even when the paresis is mild (as demonstrated by limb movement made at the request of an examiner), neglect patients frequently fail to use the limb to assist themselves when balancing or during other activities. This failure of spontaneous limb use in the face of a demonstrated ability to carry out specific behaviors when they are requested by an examiner is termed learned nonuse in the context of CI therapy. In the neglect literature, this is commonly termed “motor neglect.” The term is used because the deficit is viewed to arise as a unilateral deficit of purposive limb activation (Sampanis and Riddoch, 2013). However, it is noteworthy that (1) the neglect literature seldom contrasts this phenomenon with learned nonuse, and (2) accordingly the possibility of the deficit arising as a result of behavioral conditioning, as opposed to being an unlearned disorder, is not routinely entertained.

One of the primary reasons for the efficacy of CI therapy is that it overcomes learned nonuse. The apparent presence of this phenomenon in unilateral neglect is an additional reason to suggest that a CI therapy approach to the treatment of unilateral neglect is worth attempting, even if the initial deficient unilateral activity may not have been learned. The possibility of treating motor neglect with CI therapy has been previously raised, although not in depth (Punt and Riddoch, 2006). The approach might involve intensive massed practice of both a variety of motor aiming behaviors, as well as visual scanning exercises and other strategies for the remediation of the visual where perception problem. The training, as in CI movement therapy, would also include shaping and many elements of the behavioral TP. Insurance reimbursement would not be available for 36–40 h of therapy for unilateral spatial neglect. However, the initial question is whether this approach is efficacious. Questions of pragmatic import are of course of significance, but they do not arise until efficacy is demonstrated. At that point, it becomes worth determining how treatment can be modified to make it available for reimbursement.

There have been at least three studies in which conventional CI movement therapy has been used to reduce unilateral spatial neglect, two in which CI movement was used alone (Bollea et al., 2007; Welfringer et al., 2013), and one in which conventional CI therapy was used in conjunction with patching of the eye opposite to the neglected visual field (Wu et al., 2013). The results reported were good. This, however, is not the procedure being advocated here. The proposed objective would be to overcome both the visual aiming and “perceptual where” deficit. Conventional CI Movement therapy would have a significant role in the suggested therapeutic approach, but so would prism adaptation training and one or more procedures to improve “visual where” perception. Optimally, extended practice, shaping, and a visually appropriate TP would be embedded in the combined approach.

#### Visual field defects due to cortical injury

Stroke results in visual field deficits in approximately 25% of cases (Kerkhoff et al., 1994). The consequent cortical blindness often involves a homonymous hemianopia, in which half the visual field is affected in both eyes. This kind of visual deficit also occurs after TBI, brain resection, and other types of post-chiasmic damage. It often results in serious impairments in the activities of daily life, such as in reading, driving, and visual exploration (Zihl and Von Cramon, 1985); this can have pervasive disabling consequences for everyday activities and importantly in being able to maintain appropriate employment. The extent of a patient’s awareness of the visual field deficit can be shown on formal testing to vary with the amount of attention directed at the visual environment along with the kinds of stimuli that are presented (e.g., faces; Williams and Gassel, 1962; Gassel and Williams, 1963).

Over the last several decades a substantial number of studies have been carried out demonstrating that this type of deficit can be reduced by appropriate training techniques. Four different general strategies have been used, not only with cases of partial cortical blindness, but also in cases of retinal and optic nerve damage.

(1) Saccadic eye movement training. In this method patients are trained to make large eye movements into the blind hemifield (Kerkhoff et al., 1992, 1994; Zihl, 1995; Pambakian et al., 2004). In the procedure employed by Kerkhoff and coworkers, training was given at least once daily for 30 min, 5 days per week for 4–12 weeks. This strategy led to considerable success in the performance of visual ADL including an impressive return to part-time work in 20 of 22 patients (Kerkhoff et al., 1994). In that study actual restitution of part of the scotomatous visual field occurred in only 54% of the patients. However, the patient’s mean visual search field size increased a mean of approximately 20^∘^, an effect that persisted for at least 3 months and is presumably the basis for the substantial improvement in visual ADL. Surprisingly, this mode of rehabilitation training has not been employed as much as seems warranted.
(2) Visual restoration training. The largest amount of research effort has been directed toward this approach. Zihl and Von Cramon (1979, 1985) developed a procedure which involved stimulating the border area between the blind and intact visual fields with spots of light by perimetry. This procedure was computerized by Kasten et al. (1998, 1999) and was subsequently employed extensively by Kasten, Sabel, and their coworkers among others (cf. Mueller et al., 2007; Bergsma et al., 2011). While there has been some question in the literature as to whether this treatment approach produces a true enlargement of the visual field (Balliet et al., 1985; Reinhard et al., 2005) or even whether it has a beneficial effect (Horton, 2005a,b; Plant, 2005; Roth et al., 2009b), the burden of evidence is that vision restoration therapy is an effective means of reducing the blind field by a mean of 5^∘^ (Kasten et al., 1998) to 6^∘^ (Sabel et al., 2013).
(3) Elaboration of blind sight/covert recognition. It has long been known that the blind field in partial cortical blindness is not completely unresponsive (Bard, 1905; Riddoch, 1917). Despite reported blindness, patients can correctly report the presence of a stimulus in the blind field at above chance levels when required to choose in a forced choice paradigm. This phenomenon was termed blindsight by Sanders et al. (1974), though the term covert recognition has greater generality since the phenomenon occurs in other sensory modalities as well. The clearest early example of the extent to which training can elaborate blindsight into a useful basis for guiding behavior is the research of Humphrey (Humphrey, 1970, 1974) who worked with the monkey Helen following bilateral occipital ablation. After extensive training, Helen could run straight to a piece of food placed at a distance of 2.5 m. While training can bring blindsight to functional relevance in some humans, many patients show no evidence that this has occurred. With sufficient training however, Stoerig (2008) reports that practiced subjects can use this ability in daily life to ride bicycles, play ping pong, and even have varying degrees of conscious vision, the latter in the apparent absence of V1 activation (Rausch et al., 2000; Kleiser et al., 2001). The key appears to be the amount and nature of training given.
(4) Global training to expand the useful field of view (UFOV). Several training protocols have been employed to increase visual attention and processing speed, particularly the UFOV protocol developed by Ball and colleagues (Ball et al., 1991, 2002). Green and Bavelier (2003) have used action video game playing to enhance a range of visual skills including speed of visual processing, ability to track several objects at once (Green and Bavelier, 2006), and improved spatial resolution of visual processing as indicated by decreased distraction by visual “crowding” (Green and Bavelier, 2007). The training protocols used by Ball and colleagues and Green and Bavelier might well be used with profit with patients after brain injury.

In summary, the literature unequivocally indicates that visual deficit after brain damage can be reduced by the application of appropriate techniques. The successes achieved in the rehabilitation of visual function are certainly impressive. However, the literature also suggests that the amount of remediation that is currently being achieved can be substantially enhanced.

By far the largest amount of work to date has been carried out with various forms of vision restoration therapy, where the size of the blind field is reduced by perimetric stimulation of the border area. The current status of this area resembles where the field of motor rehabilitation after CNS damage was in the 1990s. The most frequently employed therapies at that time, such as neurodevelopmental therapy (NDT) and proprioceptive neurofacilitation (NPF), were successful in producing changes in movement in the clinic, but the focus of the therapeutic work was on producing the best movement the participant was capable of on demand in the presence of the therapist; for example, how far the patient could raise a more affected arm above shoulder level or maintain balance when subjected to a perturbing force. There was a reduced focus on how movements and movement adjustments could be used in functional activities (e.g., folding a towel, picking up a glass of water, bringing it to the mouth and drinking) when this might be requested by a therapist, and little or no attention was paid to whether the improved ability to make the trained movements generalized to the life situation. While it is incontrovertible that improving the quality of impaired movement or of making the performance of previously abolished movements again possible is important, it is also true that if the improvement on the impairment level does not lead to improved capability of carrying out functional activities in the clinic and then translation of this improved capacity to ADL in everyday life, the improvement in function has no pragmatic import. Unless this final step is taken to enhance independence and quality of life, one might argue that the improvement observed in the clinic is primarily of academic interest. The field of vision restoration training has progressed past that point. It has been shown that this therapeutic approach improves health-related quality of life and visual ADLs (Mueller et al., 2003; Sabel et al., 2004; Gall et al., 2008; Roth et al., 2009b). However, the improvements do not take place in all visual ADL areas and while the correlations between changes in field size and improvements in certain types of visually guided real-world behavior are significant, they are small. Moreover, the beneficial effects of this treatment on behavior have not been universally accepted (Horton, 2005a,b; Plant, 2005). For example, in a RCT, saccadic eye movement training was shown to be superior to a form of visual restoration therapy for improving exploration toward the blind hemifield (Roth et al., 2009a,b). In addition, fully one-third of patients given vision restoration training show no enlargement of the visual field. In comparison, CI movement therapy produces a clinically significant increase in the use of a more affected arm in the life situation. In addition, approximately 97% of patients improve a clinically significant amount (>10% of the full scale of the MAL: van der Lee et al., 2004; Taub et al., 2005; Dromerick et al., 2009). The only participants not reaching this level of success were those who explicitly rejected the therapeutic procedures after study enrollment.

The success of the visual rehabilitation of cortical blindness has been real and important. The question remains, however, whether the magnitude of the restitution could be greater, especially in terms of generalization to everyday activities in the life situation. Areas that could be explored in this regard are as follows:

- By and large, almost all the visual treatment protocols rely on repetitive practice. Though this is often carried out for 50–70 h distributed over months, no attempt is made to train by shaping, that is, to keep a person trying to improve on their personal best performance in small increments. The closest to this procedure occurs in active video game playing, though this approach has not yet been used in cases of partial cortical blindness. For healthy subjects the procedure is indirect, appearing to be inherent in the game being played. In cases of cortical blindness it would probably be of additional value to add instructions by the experimenter and formal shaping procedures.
- There has been some attention paid to relating the therapeutic gains achieved in the laboratory to improvement in visual ADLs in the clinic, but there does not appear to have been any explicit effort made to increase function in the real world, as by the use of techniques similar to those in the CI therapy TP (Taub et al., 2013b).
- There has been little focus on retention of treatment gains. In most studies there is no attempt to obtain information in treatment follow-up.

Given the impressive gains that have been made in visual rehabilitation to date, it could be that attention to these factors could yield a robust increase in the real-world relevance of the existing methods. In addition, it might be of considerable value to expand the training sessions somewhat so that two or more of the techniques described above could be combined. An objection might be that a combined approach would not be clinically feasible or acceptable to patients. However, the prior question might be whether a combined approach confers any objective improvement over use of a single technique. If that is the case, work could later be carried out to streamline a protocol; less important elements could be eliminated so that a more therapeutically potent procedure could be designed that could both be carried out in an acceptable period of time and that was accessible to an appropriately large number of patients.

## GENERAL MECHANISMS UNDERLYING EFFICACIOUS REHABILITATION OF FUNCTION AFTER BRAIN INJURY

### OVERCOMING LEARNED NONUSE REVISITED: SENSORY NONUSE AND DIMINISHED NEURAL CONNECTION STRENGTHENING

As described in an earlier section of this paper, the development of CI Movement therapy began with work with monkeys who had received a surgical abolition of sensation from one forelimb. Subsequently, the deafferented monkeys made no purposive use of that extremity though motor innervation remained intact. Sherrington explained this puzzling phenomenon on the basis of the interruption of all same-segment reflex arcs from the affected limb. This experiment formed an important basis of Sherringtonian reflexology, a dominant position in neuroscience for the first three-quarters of the twentieth century. It was based on the belief that spinal reflex arcs constituted the basic building blocks upon which purposive or voluntary movement was based. However, work from approximately 1960–1980 showed that a deafferented primate forelimb could be converted into a limb that, while not normal, could be used for a very wide variety of purposes by training of the deafferented extremity or restraint of the intact forelimb (Taub, 1977, 1980). In ambulation, for example, it was not easy for a casual observer to discriminate between an intact and a deafferented forelimb. The reflexological or peripheralist position could therefore not be correct. As a replacement, the learned nonuse (LNU) mechanism was suggested (Taub, 1977, 1980). It was held that during the early post-injury period, spinal shock rendered the segmental motor apparatus fully or partly inoperative so that the affected limb really could not be used. During this period the monkey had to learn compensatory motor patterns to carry out the basic primate ADL. The compensatory activities, primarily the use of the intact forelimb to do the work of both, involved a degraded, inefficient pattern of coordination, but since it was at least partially successful, it was reinforced, strengthened, and through repeated use overlearned. Subsequently, the spinal shock resolved spontaneously and use of the deafferented limb would have become possible, but because of the overlearning, termed learned nonuse, the newly acquired ability to use the deafferented limb was masked. Moreover, even in the chronic phase the absence of sensation in the limb reduced the afferent drive of the segmental motor apparatus, rendering movement of the limb more difficult, and thereby providing a contemporaneous basis for maintaining the LNU. Training, especially by the behavioral technique of shaping, or prolonged restraint of the intact limb forced use of the deafferented limb by increasing the motivation to use it, thereby overcoming the LNU. However, if simple repeated practice of a learned movement were carried out as in a conditioned response situation (Knapp et al., 1963; Taub and Berman, 1963; Taub et al., 1965, 1966) rather than by administering training by a shaping sequence or if the restraint was removed too soon after the appearance of purposive movement of the deafferented limb, the animal immediately reverted to nonuse of the affected extremity. The strongly learned habit of nonuse of the deafferented limb simply overcame the insufficiently or weakly learned habit of using that limb produced by the repeated repetition of a simple movement in a conditioned response situation or by an insufficiently long period of intact limb restraint. Using the affected limb still required considerable effort due to the incompletely recovered afferent drive. The LNU formulation was confirmed in two experiments (Taub, 1977, 1980). A long-term reversal of LNU could be achieved by shaping or prolonged restraint. In effect, a weakened habit or alternately a weakened neural drive had been strengthened sufficiently to make spontaneous use of the deafferented limb in the animal’s life situation possible on a long-term basis. One could also say that the animal’s ability to attend to use of the deafferented extremity had been increased or alternately weak or DNCs had been strengthened.

The first application of the CI Movement therapy protocol to humans was to patients with chronic stroke. However, as noted earlier, the LNU formulation predicted, in effect required, that the CI therapy approach apply to other types of damage to the CNS. This was subsequently confirmed for the upper extremity in work with patients with TBI, MS, cerebral palsy, other childhood motor disorders with a variety of etiologies (brain resection including hemispherectomy, TBI, different congenital brain malformations), and for the lower extremity after the same types of CNS damage and fractured hip. A CI therapy approach has also been shown to be effective for aphasia after stroke. In the present context of the rehabilitation of visual deficits, the latter is of particular interest since in aphasia it is not the motor component of speech that is affected (dysarthria), but rather the linguistic aspect. This then is a clear example of the CI therapy training approach being effective for the rehabilitation of a non-motor function after brain damage. It was also noted above that the work of Levi and Polat and their respective coworkers showed that a CI therapy-type approach was successful in the treatment of amblyopia. The content of the methods applied to each of these different functional deficits vary substantially, but the present contention is that the principles of effective rehabilitation might be the same for all of them. It is proposed herein that a CI therapy approach would also be effective for reducing unilateral visual neglect, somatosensory, and auditory deficit after stroke or other brain injury and for improving vision in partial or even complete cortical blindness.

**FIGURE 8:**
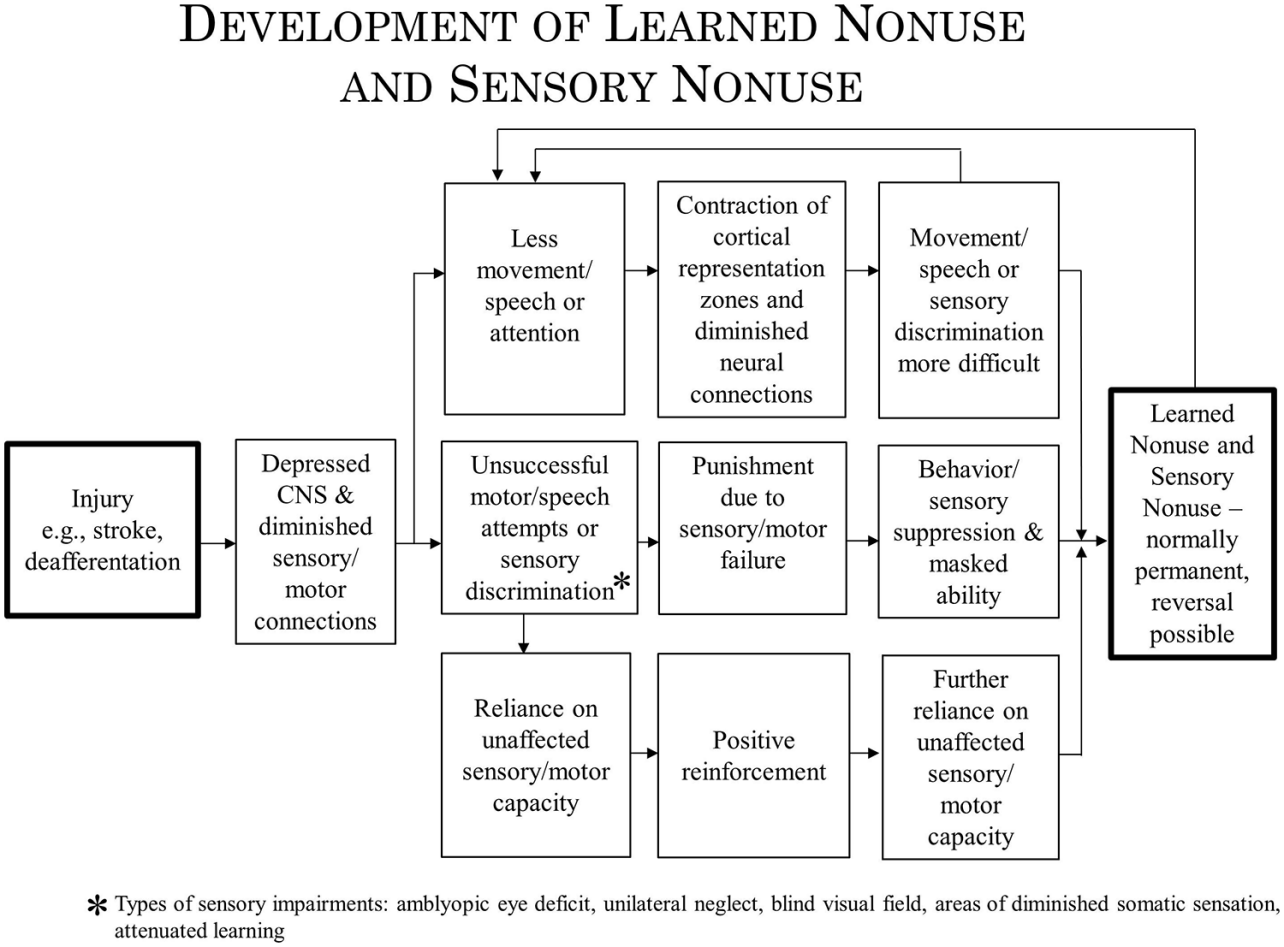
**Schematization of the development of learned nonuse and sensory nonuse**.

**FIGURE 9:**
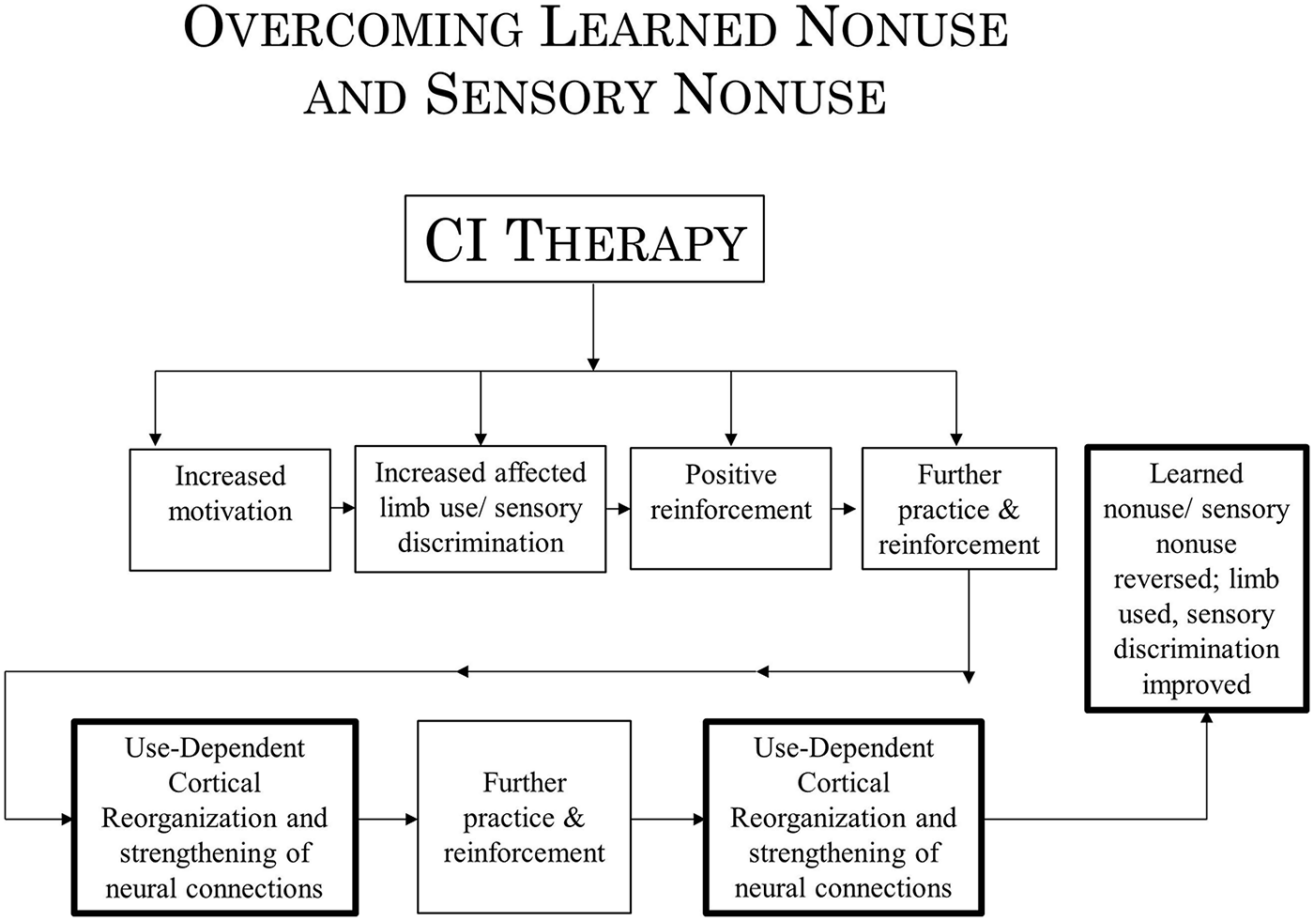
**Schematization of the process by which CI therapy overcomes learned nonuse and sensory nonuse**.

The question arises as to why all of these disparate functional deficits should be responsive to the same rehabilitation approach. On the level of mechanism, is there some commonality that would explain why such different aspects of impaired organismic function should be responsive to the same general principles of treatment, if that turns out to be the case. CI movement therapy is efficacious for motor deficits in part because it overcomes learned nonuse. This was characterized above as involving a strengthening of habits weakened by injury, which reduces on a physiological level to a strengthening of weakened or DNCs (e.g., Liepert et al., 2000a). Another way of stating the general principle is that the organizing concept is the manipulation of attention by appropriate methods of training. The individual is taught to attend to previously ignored or weak aspects of the motor repertoire or sensation. In each case the effective elements would be repetitive practice, training by shaping, discouraging inefficient compensatory patterns of motor coordination or perception, and TP techniques to integrate the newly developed motor or sensory/perceptual ability into the activities of everyday life. The result is that weak or DNCs are strengthened or increased. It is proposed that this would be as much the case for sensory function after CNS damage as it is for motor function. In the case of sensory deficits one might speak of sensory nonuse rather than learned nonuse. Both sensory nonuse and learned nonuse would be related to the more general phenomenon of the presence of DNCs compared to the stronger or more numerous neural connections that existed before brain damage, or nonuse of a function, or both. The rehabilitation of function in either sensory or motor systems could then be considered to involve diminished neural connection strengthening (DNCS). The term sensory nonuse is not meant to convey any connotation related to conscious choice or conscious awareness; that is, that a person has access to a given type of sensory input but chooses to ignore it. It is meant rather to suggest that the information associated with an afferent input is present in some form in the nervous system, but it is not responded to by an individual either because the signal is too weak to rise above a given threshold or because the signal has fallen below a threshold that would enable it to balance a countervailing sensory input that captures attention and response tendencies. Learning would be important in setting those thresholds. The development of learned nonuse and sensory nonuse and the process by which they can be overcome is schematized in **Figures 8** and **9**, respectively.

There are clearly several mechanisms that could lead to diminished or weakened neural connections. One that would be most prominent in the development of learned nonuse would be based on the loss of neural excitability following damage to the brain; this would lead to nonuse of an affected extremity which through learning would persist even when the excitability of the neural connections returned through spontaneous recovery, at least, partially, so that use of the affected limb was potentially possible. Another mechanism would involve loss of a portion of the neural connections that normally support a function due to brain damage, rendering the reduced number of connections that remain incapable of supporting that function. Input from the remaining connections would then simply be ineffective or disregarded unless the remaining connections were strengthened or supplemented by the application of an appropriate rehabilitation technique. Future research might test these two and other possible mechanisms.

### CORTICAL REORGANIZATION REVISITED: BRAIN STRUCTURE REPURPOSING

In the seminal original research of M. Merzenich and coworkers, structural brain remapping in new world monkeys was studied after amputation of a single digit. The enlargement of the cortical representation zones of the neighboring still-intact digits was small and was thought to be the result of the increased density of existing dendritic arborizations; this gave rise to the 2 mm rule which proposed that cortical reorganization could take place over a limit of 2 mm of cortical territory. Later Pons et al., working with the deafferented monkey of one of us (E.T.) found that the entire representation zone of the now-deafferented extremity had been “invaded” by extensions of the innervation of the still-intact face. The invaded zone was 10–14 mm in extension. Merzenich agreed that the 2 mm rule no longer represented a limitation of the extent possible for neuroplastic territorial cortical reorganization. The earlier view was seen to be an artifact of the experimental model studied, since the cortical representation zone of a single new world monkey digit is small. However, aspects of the original formulation continue to exert an influence and may limit the perceived horizon of what the limits of rehabilitation might be. In the study by Pons et al., using monkeys with a complete limb deafferentation, the authors coined the term “massive cortical reorganization” to describe their results. However, the process observed involved an intact cortical region spreading its connections over a contiguous region on the same side of the brain. Since then structural reorganization that is even more massive involving non-contiguous portions of the brain has been observed. In this laboratory, we have found a profuse structural remapping taking place after CI therapy in patients after stroke over all or a large part of ipsilesional motor areas of the brain, but also in non-contiguous contralesional cortex that normally contributes only a small portion of the innervation of the trained arm affected by stroke (Gauthier et al., 2008), cerebral palsy (Sterling et al., 2013), and MS (Mark et al., 2014). We have also obtained preliminary data strongly suggesting that after CIAT II there is a large increase of gray matter in the areas in the right hemisphere homologous to the language areas in the stroke-affected left hemisphere. In each of these cases, there had probably been an elaboration of a small but already existing source of functional innervation. However, even larger reorganizations of cortical territory have been found to occur. In individuals who have been blind since birth, both auditory (Kujala et al., 1992, 1995a,b, 1997; Alho et al., 1993; Weeks et al., 2000), and tactile (Rösler et al., 1993; Uhl et al., 1993; Kujala et al., 1995a; Röder et al., 1996, 1997; Cohen et al., 1997) stimuli come to be processed in the visual cortex. This remarkable case of territorial neuroplasticity and the profuse increase in gray matter in unexpected areas of the brain in correlation with the large improvements in motor function in response to CI therapy and the phenomena just cited for sensory areas of the brain would seem to warrant a new term descriptive of the extraordinary capacity of the brain to make territorial adjustments after brain damage to compensate for lost function. This marked capacity for territorial neuroplasticity, then, might be referred to as brain structure repurposing (BSR). The magnitude of what is possible with BSR or use-dependent cortical reorganization suggests that we do not yet know the limits of rehabilitation. The application of appropriate techniques such as the CI therapy approach applied in new areas of damaged function after brain injury and the combination of multiple techniques in this pursuit may give promise of the emergence of new vistas for rehabilitation.

To summarize, in this article the concept of learned nonuse, demonstrated to occur after single limb deafferentation in monkeys and movement and speech after stroke and other types of brain damage, has been elaborated to include the sensory nonuse that occurs in amblyopia, unilateral visual neglect, and visual field defects. According to the new formulation both learned nonuse and sensory nonuse involve a weakening of neural connections due to damage to the brain, the loss of connections, the reduced excitability of connections due to nonuse of a function, or a “shock-like” phenomenon in the early post-injury phase, or all of the above. CI therapy overcomes learned nonuse and it is proposed that it also has the potential for overcoming sensory nonuse by strengthening DNCs. Thus, DNC strengthening provides a bridge between learned nonuse/sensory nonuse on the one hand and use-dependent cortical reorganization on the other, the two mechanisms that presumably underlie the efficacy of CI therapy, which until now have been viewed as separate entities.

## CONCLUDING CONSIDERATIONS

In the two preceding sections several mechanisms are postulated as constituting the basis of CI therapy which has been shown to be efficacious for motor and speech deficits, and the potential extension of its principles to the domain of sensory deficits after brain damage or abnormal development. The mechanisms suggested are presented in general terms and are offered as the basis for hypothesis formation and further testing. The specific anatomical and physiological details of the mechanisms are intentionally left largely unspecified. They can be supplied by future research with the recognition that additional processes may be involved or that alternative processes may be operative. The future development of this line of conceptualization may be similar to the progressive process by which the learned nonuse formulation developed. This concept, not yet named, was proposed in 1968 (Taub and Berman, 1968). Over the next decade the operation of the proposed mechanism was confirmed experimentally in work with monkeys (Taub, 1977, 1980), and it subsequently provided a basis for the extension of the CI therapy methodology from monkeys to the upper extremity of humans after stroke (Taub et al., 1993), and subsequently to the many applications of CI therapy to other pathological conditions, the lower extremities, and speech. Learned nonuse started as a formulation that adequately explained the available data, and gradually acquired a more substantive standing as experiments confirmed its existence and numerous predictions that it generated were confirmed. This paper proposes that neural connection insufficiency or weakness lies at the basis of both learned nonuse in motor systems and sensory nonuse in sensory systems and that both can be overcome by a rehabilitation methodology that modifies the neuroplastic potential of the brain, a potential that persists throughout the lifespan and that enables BSR. It is suggested that this formulation explains much of the data currently available relating to efficacious treatments for motor and sensory deficits. However, the new formulation is tentative in terms of specific details and requires future experimental confirmation. The formulation is testable and it stands now at the level of development that the learned nonuse formulation originally had when it was first proposed.

## Conflict of Interest Statement

The authors declare that the research was conducted in the absence of any commercial or financial relationships that could be construed as a potential conflict of interest.
